# Extracellular nicotinamide phosphoribosyltransferase boosts IFNγ-induced macrophage polarization independently of TLR4

**DOI:** 10.1016/j.isci.2022.104147

**Published:** 2022-03-23

**Authors:** Giorgia Colombo, Cristina Travelli, Chiara Porta, Armando A. Genazzani

**Affiliations:** 1 Department of Pharmaceutical Sciences, University of Eastern Piedmont, A. Avogadro, 28100 Novara, Italy; 2 Department of Drug Sciences, Università degli Studi di Pavia, 27100 Pavia, Italy; 3 Center for Translational Research on Autoimmune & Allergic Diseases (CAAD), Università del Piemonte Orientale, 28100 Novara, Italy

**Keywords:** Biological sciences, Immunology, Transcriptomics

## Abstract

Nicotinamide phosphoribosyltransferase (NAMPT), alongside being a crucial enzyme in NAD synthesis, has been shown to be a secreted protein (eNAMPT), whose levels are increased in patients affected by immune-mediated disorders. Accordingly, preclinical studies have highlighted that eNAMPT participates in the pathogenesis of several inflammatory diseases. Herein, we analyzed the effects of eNAMPT on macrophage-driven inflammation. RNAseq analysis of peritoneal macrophages (PECs) demonstrates that eNAMPT triggers an M1-skewed transcriptional program, and this effect is not dependent on the enzymatic activity. Noteworthy, both in PECs and in human monocyte-derived macrophages, eNAMPT selectively boosts IFNγ-driven transcriptional activation *via* STAT1/3 phosphorylation. Importantly, the secretion of eNAMPT promotes the chemotactic recruitment of myeloid cells, therefore providing a potential positive feedback loop to foster inflammation. Last, we report that these events are independent of the activation of TLR4, the only eNAMPT receptor that has hitherto been recognized, prompting the knowledge that other receptors are involved.

## Highlights

• eNAMPT triggers an M1-skewed transcriptional program
• eNAMPT effects are independent from the extracellular enzymatic activity
• eNAMPT selectively boosts IFNγ-driven transcriptional activation
• eNAMPT events are independent by the activation of TLR4

## Graphical abstract

**Figure:**
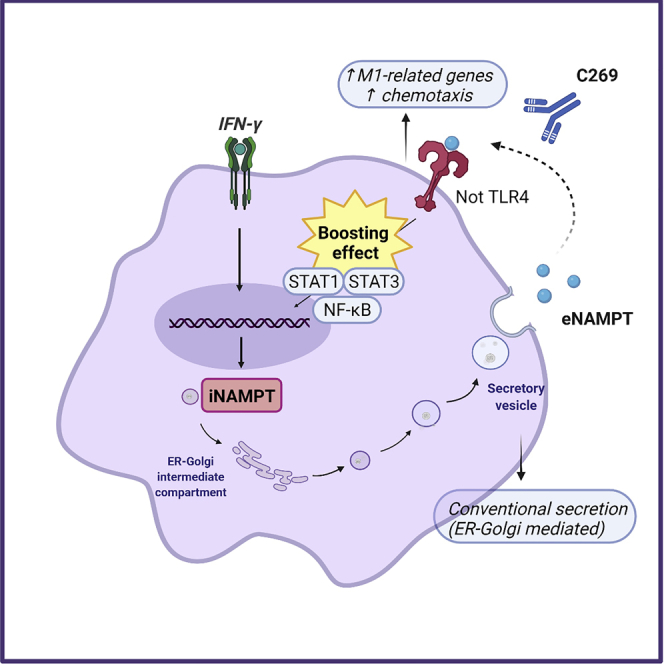

## Introduction

Intracellular nicotinamide phosphoribosyltransferase (iNAMPT) has received significant attention over the years, as it represents the cytosolic rate-limiting enzyme of the NAD salvage-pathway in mammals and catalyzes the synthesis of nicotinamide mononucleotide (NMN) from nicotinamide (NAM, vitamin B3, or PP) and 5-phosphoribosylpyrophosphate (PRPP) (Garten et al., 2015). NAMPT has also been shown to be a secreted protein. Indeed, extracellular NAMPT (eNAMPT) is the same protein that was described as pre-B-cell enhancing factor (PBEF) for its ability to synergize with interleukin-7 (IL-7) and stem cell factor, increasing the number of pre-B-cell colonies, and as visfatin, a cytokine first described as released from adipose tissue (Fukuhara et al., 2007; Revollo et al., 2007; Samal et al., 1994). A number of groups, including ours, have shown that eNAMPT can be secreted by immune cells (Audrito et al., 2015; Curat et al., 2006; Halvorsen et al., 2015; Laudes et al., 2010) as well as by other cell types in a classic manner (Grolla et al., 2015; Tanaka et al., 2007), and recently it has been shown that eNAMPT can also be present in secreted microvesicles (Grolla et al., 2015; Yoshida et al., 2019). How eNAMPT exerts its extracellular functions has not been fully elucidated (Camp et al., 2015). Van der Bergh et al. proposed a direct binding to CCR5 in macrophages and PBMCs *in vitro* (Van den Bergh et al., 2012), and we have indeed confirmed that eNAMPT may have an antagonistic role on this receptor, although it does not appear to be the principal pathway by which it exerts most of its actions (Torretta et al., 2020). Controversially, a different group brought evidence that eNAMPT might instead also have agonist properties, acting on muscle stem cells and promoting muscle regeneration (Ratnayake et al., 2021). On the other hand, it has also been shown that eNAMPT leads to TLR4 activation. Evidence for this comes from surface plasmon resonance (Camp et al., 2015; Managò et al., 2019), from an effect on human macrophages, and from an antagonistic effect of a TLR4 antibody (Camp et al., 2015; Managò et al., 2019).

Different groups have pointed out that eNAMPT modulates different myeloid cell activities in a context-specific manner (reviewed in (Travelli et al., 2018)). eNAMPT promotes M1-polarization in both murine bone-marrow-derived macrophages and in human monocyte-derived macrophages, determining an increase of iNAMPT and the secretion of tumor necrosis factor alpha (TNFα) and IL-6 (Bermudez et al., 2017; Halvorsen et al., 2015; Moschen et al., 2007; Wu et al., 2018). However, in a tumor setting, which alters myelopoiesis and functional skewing of monocytes, eNAMPT further enhances the expression of immunosuppressive M2 genes such as IL-10, IDO, CD206, and CD163 (Audrito et al., 2015). Moreover, eNAMPT appears to foster macrophage phagocytic activity (Yun et al., 2014) and to favor macrophage migration by inducing the expression of matrix metalloproteinases (Dahl et al., 2007). Despite this encouraging evidence, a thorough characterization of the actions of eNAMPT on macrophages is lacking.

Although the mechanisms underpinning eNAMPT activity remain largely unclear, it is well established that eNAMPT participates in the pathogenesis of several inflammatory conditions, as demonstrated by the beneficial effects of its neutralization in experimental models of colitis (Colombo et al., 2020) and inflammatory lung injury (Garcia et al., 2021; Quijada et al., 2021).

Given that macrophages are pivotal orchestrators of both initiation and resolution of inflammation, we undertook a full investigation of the effects of eNAMPT on primary murine peritoneal macrophages (PECs), an approach that has the advantage of giving insights on the physiological role of this protein using primary cells. Our data show that eNAMPT promotes macrophages-driven inflammation mainly in a Toll-like receptor 4 (TLR4)-independent manner. Specifically, we found out that eNAMPT (1) promotes chemotactic recruitment of inflammatory cells, (2) activate macrophages to express an M1-skewed transcription program, (3) boosts IFNγ-driven macrophage activation by enhancing STAT1/3 activation, and (4) is strongly released in response to IFNγ treatment, thereby providing a potential positive feedback loop supporting exacerbation of inflammation.

## Results

### eNAMPT is an M1-skewing stimulus

**Figure 1:**
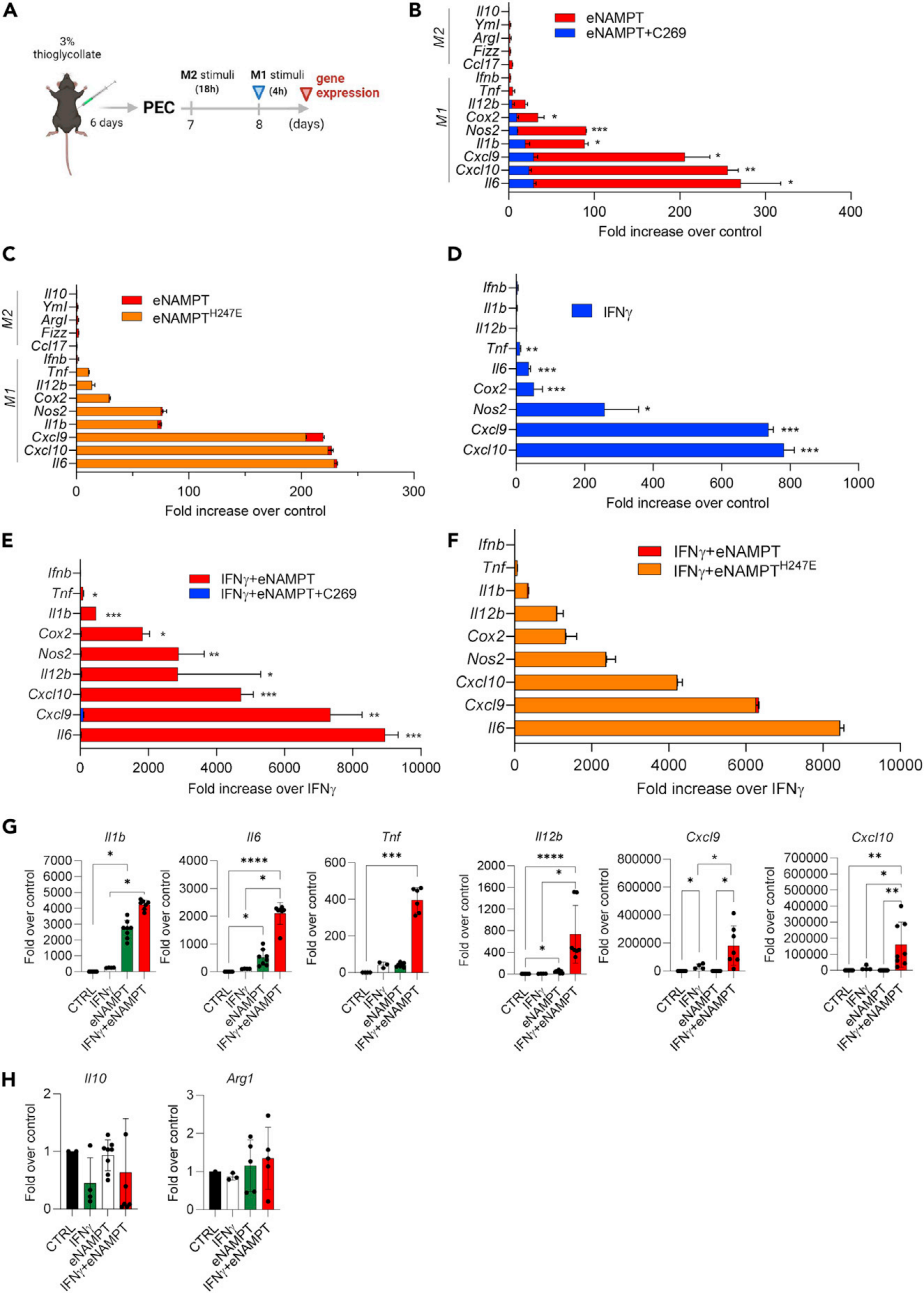
eNAMPT skews murine and human macrophage toward M1 polarization (A) Representative scheme of experimental plan (created with BioRender). (B) Gene expression changes of the indicated genes in response to eNAMPT (500 ng/mL) or eNAMPT and C269 (10 μg/mL) in murine PECs. Mean ± S.E.M. of 7 independent experiments. (C) Gene expression changes of the indicated genes in response to eNAMPT (500 ng/mL) or H247E NAMPT (500 ng/mL) in murine PECs. Mean ± S.E.M. of 2 independent experiments. (D) Gene expression changes in response to IFNγ (200 U/mL) in murine PECs. Mean ± S.E.M. of 7 independent experiments. (E) Gene expression changes in response to eNAMPT (500 ng/mL) and IFNγ (200 U/mL) in the presence or absence of C269 (10 μg/mL) in murine PECs. Mean ± S.E.M. of 3 independent experiments. (F) Gene expression changes in response to eNAMPT (500 ng/mL) and IFNγ (200 U/mL) and to H247E NAMPT (500 ng/mL) and IFNγ (200 U/mL) in murine PECs. Mean ± S.E.M. of 2 independent experiments. (G and H) Gene expression changes in human monocyte-derived macrophages treated with eNAMPT (500 ng/mL) and/or IFNγ (200 U/mL) for 24 h. Mean ± S.E.M. of 3 independent experiments. p value: ∗p < 0.05; ∗∗p < 0.01; ∗∗∗p < 0.001∗∗∗∗p < 0.0001.

To unravel the effect of eNAMPT on macrophages, according to the guidelines (Murray et al., 2014), we stimulated PECs with murine recombinant eNAMPT (500 ng/mL, endotoxin levels less than 0.1 EU/mL). To ascertain the specific role of eNAMPT on gene expression, cells were treated in the presence or absence of C269 (10 μg/mL), an eNAMPT-neutralizing monoclonal antibody that we have recently generated and validated (Colombo et al., 2020). Given the different kinetics of M1 and M2 gene induction, we analyzed transcript levels after 4 and 18 h, respectively (Figure 1A). In comparison with untreated PECs, qPCR results showed that eNAMPT induced all inflammatory M1-related genes tested, including *Il6*, *Il1b*, *Cxcl10*, *Cxcl9*, *Nos2*, *Cox2*, *Tnf*, and *Il12b*, whereas neither the anti-inflammatory cytokine *Il10* nor the M2(IL-4)-associated genes were modulated (Figure 1B). As shown in Figure 1B, C269 (blue bars) abrogated the effect of eNAMPT demonstrating the specificity of the effect. To evaluate whether the effect of eNAMPT could be attributed to its enzymatic activity, we next stimulated PECs with eNAMPT^H247E^, a mutant that has been shown to lose the catalytic activity (Wang et al., 2006). eNAMPT^H247E^ was able to induce M1-associated genes (Figure 1C) to the same extent as wild-type eNAMPT, conclusively proving that the extracellular enzymatic activity is dispensable for macrophage skewing.

Given that in several inflammatory conditions NAMPT has been shown to increase and act as an exacerbator of inflammation (Travelli et al., 2018), we explored the crosstalk between eNAMPT and other inflammatory stimuli. We treated PECs with interferon gamma (IFNγ), lipopolysaccharide (LPS), IL-6, IL-1β, granulocyte-macrophage colony-stimulating factor (GM-CSF), and IL-4 either as single stimuli (Figure 1D) or in combination with eNAMPT (Figures 1E and S1A–S1E). The results indicated that eNAMPT strongly enhanced the expression of IFNγ and LPS-induced genes (Figures 1E and S1A). The pattern of potentiation was not identical between IFNγ and LPS, although in both settings *Il6* was the most upregulated gene over the respective stimulus. On the contrary, IL-6, IL-1β, and GM-CSF responsive genes were not further induced by eNAMPT (Figures S1B–S1D). We confirmed the specific boosting effect of eNAMPT by using C269, which completely prevented the increased expression of IFNγ-induced genes (Figure 1E). Moreover, we verified that the synergism is maintained also with catalytically inactive eNAMPT^H247E^ mutant (Figure 1F; see Figure S1F for residual enzymatic activity of the mutant), confirming that also this phenomenon is not dependent on the enzymatic activity of the protein. We next investigated whether these observations could have relevance to humans by evaluating the effect of eNAMPT on human macrophages differentiated *in vitro* from monocytes of healthy donors. Of the selected gene panel, we confirmed that human recombinant eNAMPT alone significantly increased *Il6, Il1b*, and *Il12b* and in combination with IFNγ further enhanced the expression of IFNγ-induced genes *Cxcl9* and *Cxcl10* (Figure 1G). The combination of eNAMPT and IFNγ also potentiated the induction of the inflammatory genes *Il6*, *Il1b*, *Il12b*, *and Tnf*, whereas, as expected, no effect by eNAMPT, IFNγ, or the combination was observed on *Il10* and *Arg1* expression (Figures 1G and 1H).

Overall, these data indicate that eNAMPT is a cytokine endowed with selective M1-skewing activity and with a potent boosting activity on IFNγ-induced activation in both murine and human macrophages.

### Characterization of the M1 signature elicited by eNAMPT

**Figure 2:**
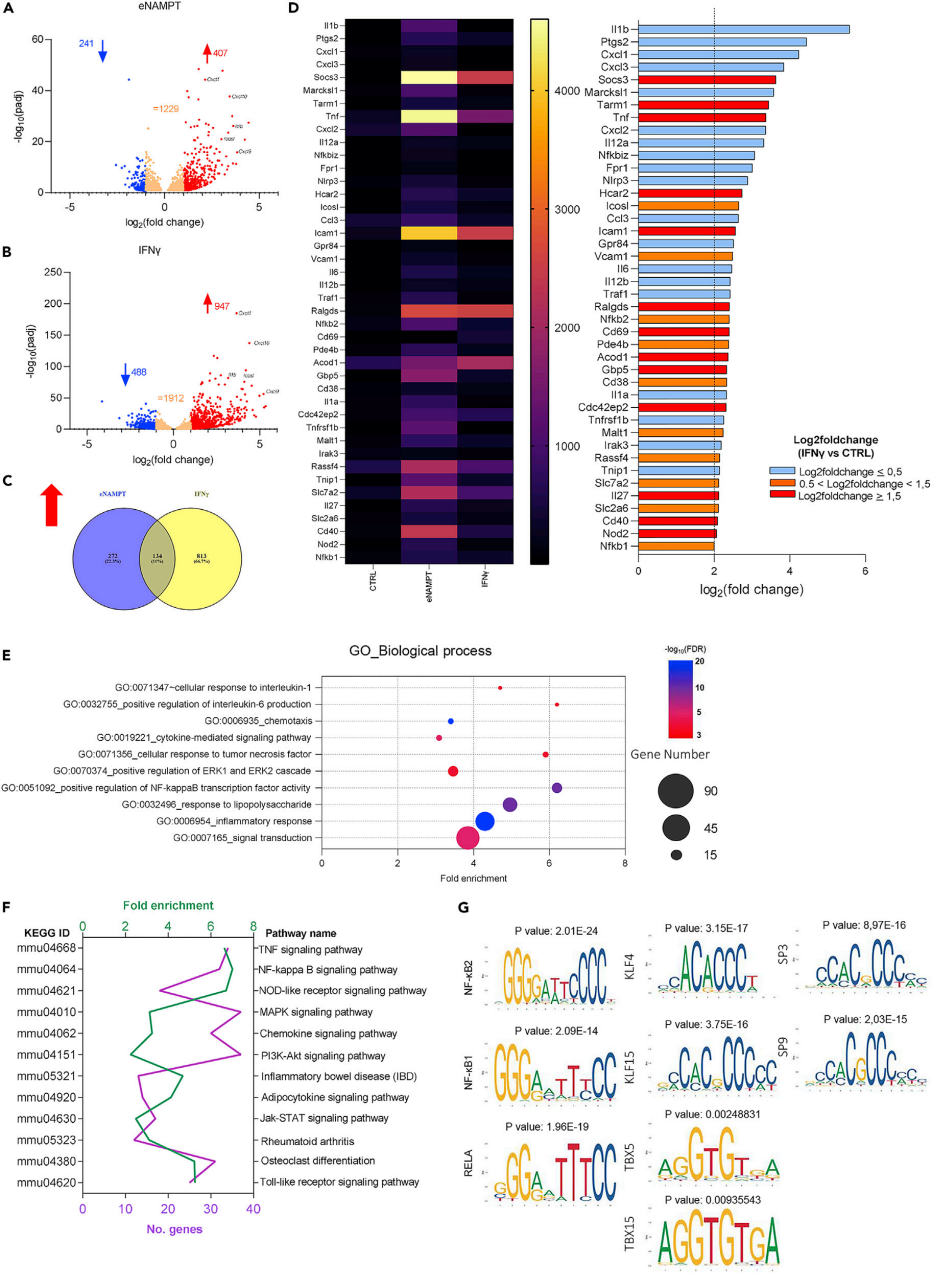
eNAMPT triggers a unique M1 signature (A and B) Volcano plot of the differentially expressed genes by eNAMPT (500 ng/mL) or IFNγ (200 U/mL), respectively, using RNAseq analysis on PECs (n = 5 replicates/condition); FDR ≤ 0.05; (C) Venn diagram of the relationship between eNAMPT- and IFNγ-regulated genes (FDR ≤ 0.05 and log2 fold-change > 1); (D) heatmap and histogram representation depicting the most upregulated genes by eNAMPT. Light blue bars represent those genes that are not regulated by IFNγ (log2-fold change < 0.5), orange represents those genes that are moderately regulated by IFNγ (0.5 < log2-fold change < 1.5), and red represents those genes that are highly regulated by IFNγ (log2-fold change > 1.5); (E) gene ontology analysis of eNAMPT-upregulated genes; (F) top 12 pathways highlighted by KEGG analysis emerging from eNAMPT-upregulated genes; number of genes annotated in each pathway (purple) and fold enrichment (green) are shown; (G) patterns of transcription factor motif enrichment within the promoters of the eNAMPT-upregulated genes.

To fully characterize the effect of eNAMPT on macrophage-polarized activation, we carried out a comprehensive analysis of the transcriptional profile of PECs by RNA sequencing. Cells were stimulated for 4 h with eNAMPT (500 ng/mL) or with IFNγ (200 U/mL), as a reference stimulus inducing classic M1-polarized activation (Adams and Hamilton, 1984). When using a log2 fold-change of at least 1 with an FDR below 0.05, eNAMPT upregulated 407 genes over control (Figure 2A). When validating a selected 20-gene set by qPCR, we found a strong correlation between the two techniques, thereby validating our findings (Figure S2A). The IFNγ-induced gene expression pattern was coherent with the literature (Das et al., 2018; Piccolo et al., 2017) and resulted in the induction (with the same cut-offs as above) of 947 genes (Figure 2B). The concordance between the two stimuli was low, with only 134 out of 1,219 genes (11%) that were significantly upregulated by both (Figure 2C). These results suggest that eNAMPT and IFNγ activate two different pathways that ultimately regulate distinct transcriptional programs. Notably, a poor superimposition was confirmed also in terms of extent of gene expression, indeed only a few genes (12 out of the 42 genes) that were upregulated by eNAMPT with at least a log2 fold-change above 2 were also upregulated by IFNγ (red bars) (Figure 2D). Although some of these differences may be attributable to the cut-offs chosen (for example, *Icosl* has a fold-change of 1.5 with IFNγ), most genes were selectively upregulated by eNAMPT (light blue bars), thereby representing an inflammatory signature that is distinct from the IFNγ ones. For example, *Il1b*, *CxCl1*, and *CxCl3* are strongly induced by eNAMPT and repressed by IFNγ (*Il1b* log2 fold-change −0.19, *CxCl1* log2 fold-change −2.44, and *CxCl3* log2 fold-change −1.0). We also analyzed the genes downregulated by eNAMPT or IFNγ. Using the same cut-offs as above, 241 and 489 genes were repressed by eNAMPT and IFNγ, respectively (Figure S2B). Again, concordance between the two stimuli was low, and the 20 most downregulated genes by eNAMPT are displayed in Figure S2C.

Figure 2E shows the top 10 most enriched pathways by eNAMPT using gene ontology (GO) analysis. As expected, there is an enrichment in inflammatory response genes, including those involved in LPS and cytokine (TNFα, IL-6, and IL-1β) responses as well as genes associated with the activation of ERK and NF-κB cascades. Interestingly, the same analysis also highlighted an enrichment in genes involved in chemotaxis. Moreover, the GO molecular function confirmed that the binding of eNAMPT to a receptor (not shown) is the most plausible mechanism whereby eNAMPT modulates gene expression. We also performed KEGG pathway enrichment analyses of the genes upregulated by eNAMPT (Figure 2F), and we corroborated the involvement of several inflammatory signaling pathways including TNF, NF-κB, JAK-STAT, MAPK, PI3K-AKT, and TLRs. We also analyzed the 241 downregulated genes, but no enrichment was found using either the GO or KEGG databases. Last, we performed predictive analysis of transcription factors driving the upregulated DEGs *via* Pscan and JASPAR. The results highlighted NF-κB, KLF, and TBX family members as the most enriched transcription factors (Figure 2G).

### eNAMPT promotes chemotaxis in a TLR4-independent manner

The above results pointed out three functional observations: (1) NF-κB appears to be an important mediator of eNAMPT responses; (2) eNAMPT responses seem to be not dissimilar to LPS responses, posing the question on whether eNAMPT acts *via* TLR4, as previously proposed (Camp et al., 2015); and (3) eNAMPT could play a role in chemotaxis. We also proceeded in analyzing the DEGs with STRING, that predicts interaction between gene products. As shown in Figure S2D, eNAMPT-responsive genes could be clustered in five networks, the three highlighted in the above points as well as IL-6 and TNF networks.

**Figure 3:**
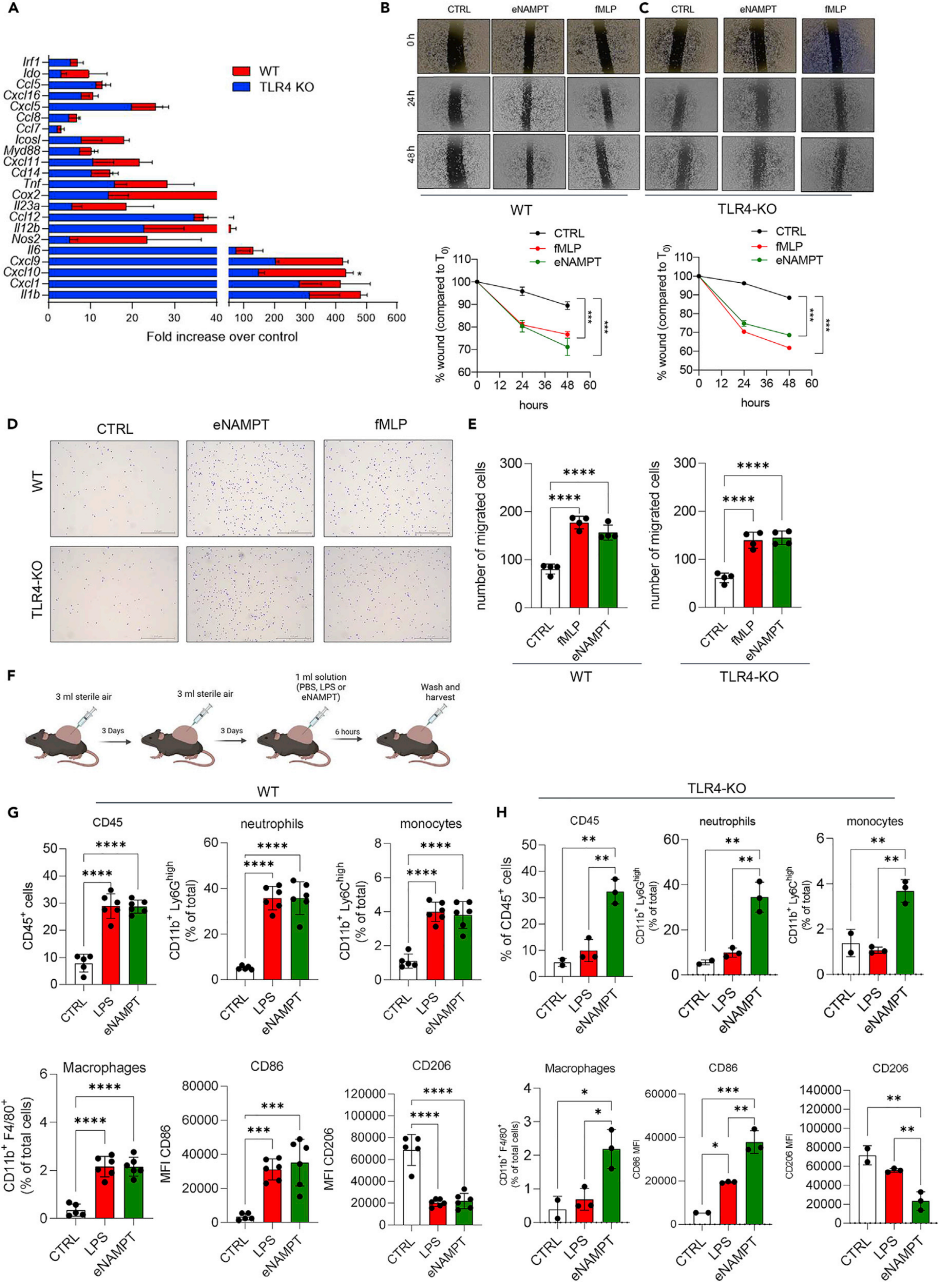
eNAMPT has pro-migratory properties *in vitro* and *in vivo*, not mediated by TLR4 (A) Gene expression changes of the indicated genes in response to eNAMPT (500 ng/mL) in PECs from wild-type (WT) and TLR4 knock-out (TLR4-KO) mice. Mean ± S.E.M. of 3 independent experiments; (B and C) representative wound healing images (top) and analysis (bottom) of PECs from wild-type and TLR4-KO mice, treated with vehicle, eNAMPT (500 ng/mL), or fMLP (1 μM). Mean ± S.E.M. of 6 determinations from 3 separate experiments; (D) representative microscopic images; and (E) quantification of PECs from WT or TLR4-KO mice migrated through transwells (Crystal violet stain, magnification 40×). Mean ± S.E.M. of 6 determinations from 3 separate experiments. (F) Representative scheme of the air pouch model; mice were treated with vehicle (PBS; CTRL), LPS (1 μg/mL), and eNAMPT (50 μg/mice). (G and H) FACS analysis of immune cells harvested from the air pouch lavage in WT and TLR4-KO mice. Mean percentage ± SEM from 3 separate experiments. p value: ∗p < 0.05; ∗∗p < 0.01; ∗∗∗p < 0.001; ∗∗∗∗p < 0.0001.

Given that it has been firmly demonstrated that NF-κB acts down-stream of eNAMPT (Camp et al., 2015; Managò et al., 2019), we did not pursue this further. We instead decided to investigate the responses of TLR4-KO PECs to eNAMPT. As shown in Figure 3A, eNAMPT triggered a similar response in wild-type and TLR4-KO PECs, whereas, as expected, LPS did not elicit any response on PECs derived from TLR4-KO mice (data not shown). Among the 22 genes evaluated, *CxCl10* only was statistically reduced in TLR4-KO PECs treated with eNAMPT. On a descriptive front, the expression of a few genes was blunted (e.g., *Nos2, Il23a, CxCl9*, or *Il12b*), and the others were virtually unchanged. These data suggest a minor contribution of TLR4 in eNAMPT-induced gene expression along with the existence of an alternative receptor for eNAMPT-driven M1 macrophage activation.

Next, to unravel the potential impact of eNAMPT on macrophage migratory behavior, we performed functional *in vitro* and *in vivo* assays. First, we carried out a wound healing assay. PECs were seeded at the concentration required to cover cell culture area, scratched and treated with 500 ng/mL eNAMPT or 1 μM of the chemotactic peptide N-formyl-methionyl-leucyl-phenylalanine (fMLP) as a positive control (Ortiz-Masiá et al., 2010). We monitored wound closure at different time points, and we found that eNAMPT and fMLP similarly accelerated wound closure compared with control (Figures 3B and 3C). Next, using a Transwell migration assay, we evaluated the chemotactic response of PECs toward eNAMPT or fMLP. The results confirmed a remarkable increase of PECs recruited in response to either eNAMPT or fMLP (Figures 3D and E).

To corroborate this chemotactic activity *in vivo*, we performed the subcutaneous air pouch model (Figures 3F–3H and S3), enabling the analysis of inflammatory cell response to local chemoattractants (Lu et al., 2020). eNAMPT (50 μg), LPS (1 μg; as a positive control), or an equal volume of PBS were injected subcutaneously, in the air pouch, and, after 6 h, cells recruited were harvested and analyzed by flow cytometry. The results showed a significant accumulation of leukocytes (CD45^+^cells) including neutrophils (CD11b^+^Ly6G^high^Ly6C^low/-^ cells), monocytes (CD11b^+^Ly6G^−^Ly6C^high^ cells), and macrophages (CD11b^+^F4/80^+^Ly6C^low/−^ cells) in the air pouches injected with eNAMPT- or LPS as compared with PBS. Moreover, macrophages showed a CD86^high^CD206^low^ phenotype that implies an M1-skewed polarized activation (Figure 3H).

To determine the potential contribution of TLR4 in eNAMPT chemotactic activity, we carried out *in vitro* migration assays with TLR4-KO PECs, and we found that eNAMPT still promoted PEC migration in both wound healing model (Figure 3C) and in Transwell migration assay (Figure 3E). In keeping with WT PECs, the effect of eNAMPT was comparable to fMLP on TLR4-KO PEC, strengthening that eNAMPT induces PEC migration in a TLR4-independent manner. Consistently, TLR4-KO mice showed an impaired recruitment of inflammatory cells in the air pouch upon LPS treatment but maintained responsiveness to eNAMPT (Figure 3H).

### eNAMPT boosts IFNγ responses in a TLR4-independent manner

**Figure 4:**
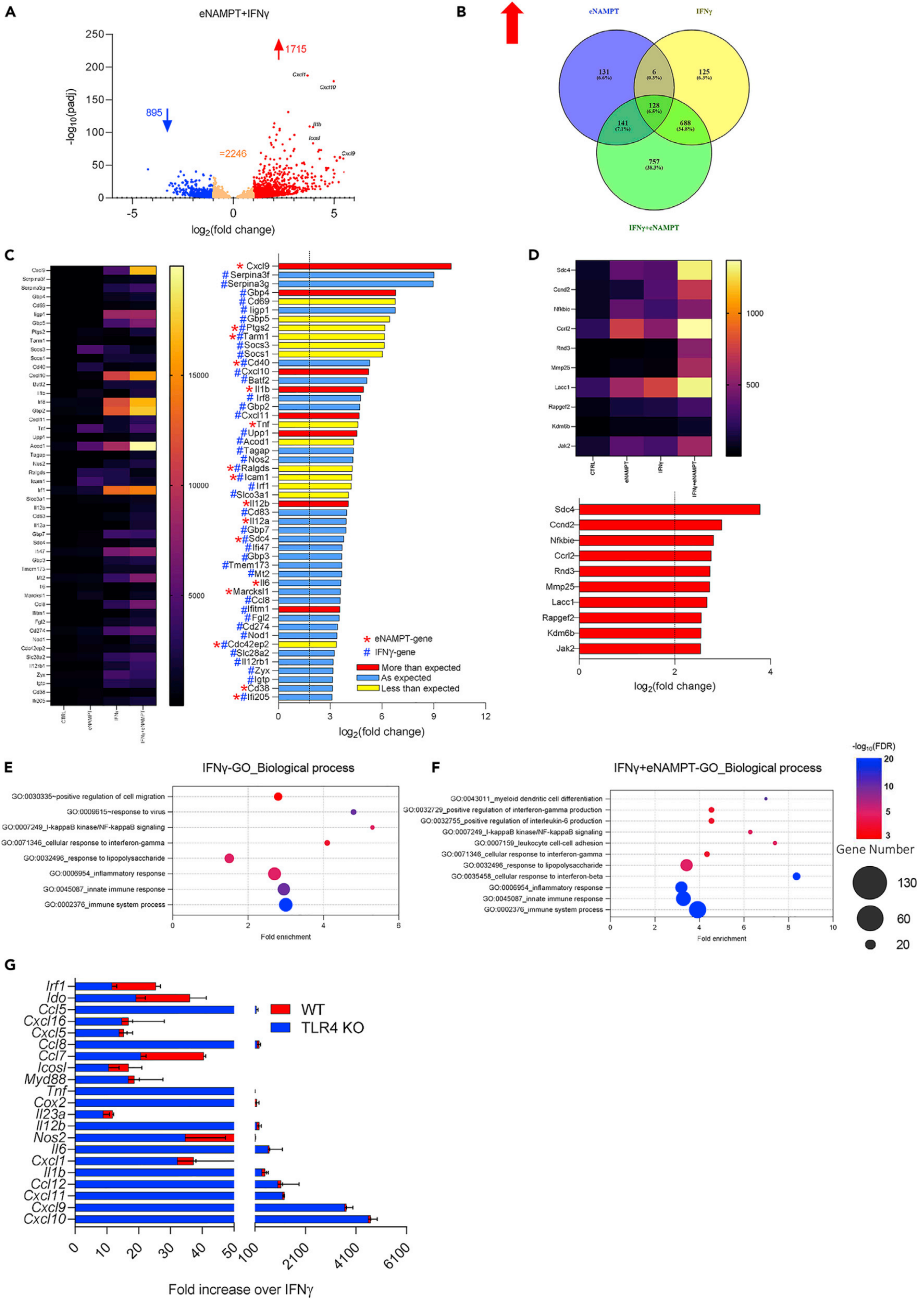
eNAMPT acts as a boosting-IFNγ response (A) Volcano plot of the differentially expressed genes by eNAMPT (500 ng/mL) and IFNγ (200 U/mL) using RNAseq analysis on PECs (n = 5 replicates/condition); FDR ≤ 0.05; (B) Venn diagram of the relationship between eNAMPT-, IFNγ-, and combination-regulated genes (FDR ≤ 0.05 and log2 fold-change > 1); (C) heatmap and histogram representation depicting the most upregulated genes by the combination; red bars represent genes with fold changes higher than expected, blue bars represent genes with fold changes as expected, and yellow bars less than expected (see “Combinatory evaluation”, Table S3). Genes are indicated with (∗) or (#) according to the dependence on eNAMPT or IFNγ stimulation, respectively. (D) Heatmap and histogram representation depicting the most upregulated genes among the 757 genes that appear only in the combination; (E and F) gene ontology analysis of IFNγ-upregulated and IFNγ+eNAMPT-upregulated genes; (G) gene expression changes of the indicated genes in response to IFNγ+eNAMPT in PECs from wild-type (WT) and TLR4 knock-out (TLR4-KO) mice. Mean ± S.E.M. of 3 independent experiments.

To get insight into the inflammatory activities of eNAMPT, we decided to explore the effect that eNAMPT exerts toward IFNγ responses (Figure 1D). As shown in Figure 4A, the co-stimulation of PECs with eNAMPT and IFNγ regulated a significantly higher number of genes (1715 genes upregulated versus 895 downregulated) than control and single treatments (Figure 2B). Also in this case, we confirmed the correlation between RNAseq and qPCR (Figure S4A). As shown in Figure 4B, most genes upregulated by eNAMPT or IFNγ as single stimuli (Figure 2C) are also upregulated by the combination. Indeed, most of the genes that are induced by IFNγ (86.1%) are still significantly induced in presence of eNAMPT, whereas approximately two-thirds of the genes (66.1%) upregulated by eNAMPT alone emerged also upon the combination. Of note, a considerable number of genes induced by the combination (44.2%) were not significantly modulated by the single stimuli, indicating that the co-presence of eNAMPT and IFNγ might activate new transcriptional programs or enhance the expression of weakly induced genes, leading to an increase of those that overcome the threshold (log2 fold-change>1; FDR <0.05). We next focused on the 50 top-ranked genes that were upregulated by the combination (Figure 4C). Among these genes, we could find a few genes that were part of the NAMPT signature (i.e., *CxCl9*, *Il1b*, *Il12b*, *Il6,* and *Marcksl1* and *Cd38*, Figure 4C), whereas most genes belonged to the IFNγ signature. Overall, the combination significantly induced a more pronounced upregulation of genes than the single agents (Figures 2D and 2E); indeed, all the genes have a log2 fold-change higher than 3 rather than 2. We also evaluated whether the effects of the combination could be additive or synergic (see “Combinatory evaluation”, Table S3), and we observed that most genes were induced in an additive manner along with a small group of genes, including *Cxcl9*, *Cxcl10*, *Cxcl11*, *Gbp4*, *Il1b*, and *Il12b*, that were synergistically upregulated. The effect of the combination IFNγ and eNAMPT is less than additive for only a few of the genes induced. Next, we evaluated the genes that were selectively upregulated by the combination of eNAMPT and IFNγ, and we found that only 10 genes (Figure 4D) showed a log2 fold induction above 2. These results suggest that the main biological functions modulated by the combination are likely associated with the genes belonging to either eNAMPT or IFNγ signatures. Therefore, we performed gene ontology (GO) analysis on the top-ranked genes, and we found out that the majority of the pathways enriched by the combination (e.g. immune system processes, cellular response to IFNγ, Figure 4F) are also typically associated with IFNγ response (Figure 4E). These results strengthened the concept that eNAMPT has a powerful boosting effect of IFNγ response. We also analyzed the 895 downregulated genes (Figures S4B and S4C), but no obvious trend was observable.

Last, we evaluated the contribution of TLR4 in eNAMPT-dependent promotion of IFNγ-induced gene expression. We observed that the response of TLR4-KO PECs to the combination of eNAMPT and IFNγ was similar to WT PECs (Figure 4G), demonstrating that eNAMPT boosts IFNγ-driven inflammatory gene expression in a TLR4-independent manner.

### The boosting effect of eNAMPT on IFNγ is mediated by STAT1/3

**Figure 5:**
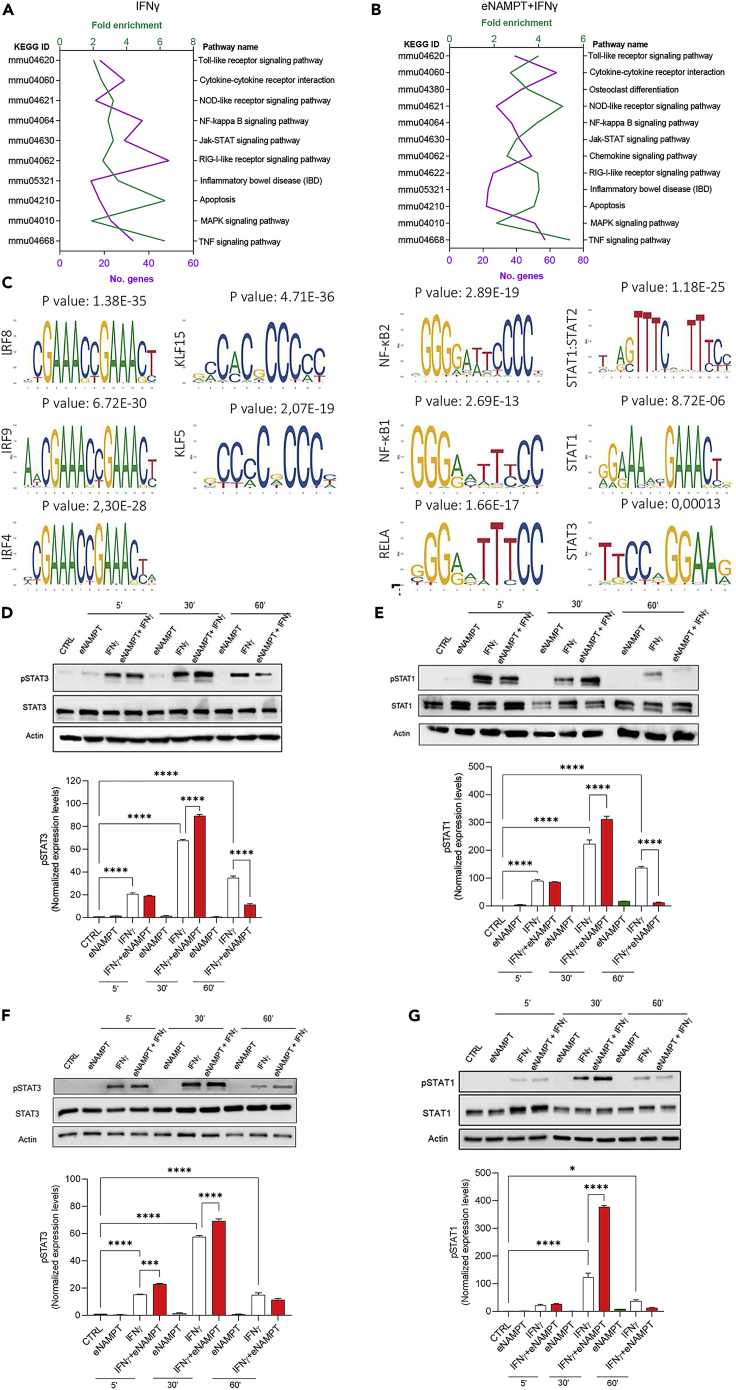
The cytokine eNAMPT enhances IFNγ-induced STAT1 and STAT3 phosphorylation in a TLR4-independent manner (A and B) Top pathways highlighted by KEGG analysis emerging from IFNγ- and IFNγ+eNAMPT-upregulated genes; number of genes annotated in each pathway (purple) and fold enrichment (green) are shown. (C) Patterns of transcription factor motif enrichment within the promoters of the IFNγ+eNAMPT-upregulated genes; (D) representative western blots of pSTAT3(Y705) and STAT3 in PECs from *WT* mice upon stimulation with eNAMPT, IFNγ, or eNAMPT+IFNγ for the indicated times and densitometric analysis. Mean ± S.E.M. of 3 independent experiments. (E) Representative western blots of pSTAT1 (Y701) and STAT1 in PECs from *WT* mice upon stimulation with eNAMPT, IFNγ, or eNAMPT+IFNγ for the indicated times and densitometric analysis. Mean ± S.E.M. of 3 independent experiments. (F) Representative western blots of pSTAT3(Y705) and STAT3 in PECs from *TLR4-KO* mice upon stimulation with eNAMPT, IFNγ, or eNAMPT+IFNγ for the indicated times and densitometric analysis. Mean ± S.E.M. of 3 independent experiments. (F) Representative western blots of pSTAT1 (Y701) and STAT1 in PECs from *TLR4-KO* mice upon stimulation with eNAMPT, IFNγ, or eNAMPT+IFNγ for the indicated times and densitometric analysis. Mean ± S.E.M. of 3 independent experiments. p value: ∗p < 0.05; ∗∗∗p < 0.001; ∗∗∗∗p < 0.0001.

We next performed the KEGG analysis of the combination dataset. As shown in Figures 5A and 5B, we observed an enrichment of the genes associated with the activation of the JAK-STAT pathway in the combination compared with IFNγ alone. A modest increase of this pathway also emerged in the eNAMPT dataset (Figure 2G). Moreover, we analyzed the putative transcription factors regulating gene expression programs *via* Pscan and JASPAR. As expected, IFNγ showed an enrichment of IRFs, NF-κB family members, and STATs (Figure S5A). The combination did not highlight any new transcriptional signatures but showed an enrichment of the transcription factors that were associated with either eNAMPT or IFNγ (Figure 5C). We also focused the analysis on the 757 genes that were upregulated by the combination only (Figure 4B). Strikingly, we observed only transcription factors (e.g., KLF and SP families) that are associated with eNAMPT signature. In contrast, STAT emerged only in association with IFNγ, either alone or in combination (Figure S5B). We therefore investigated the effects of eNAMPT on IFNγ-induced STAT activation. We stimulated PECs with eNAMPT, IFNγ, or their combination, and we analyzed the phosphorylation of STAT1, which is the main transcription factor regulating IFNγ-induced gene expression, and STAT3, which is already known to be activated by eNAMPT (Li et al., 2008) and to be a modulator of IFNγ biological activity (Qing and Stark, 2004). The results showed that the combination of eNAMPT and IFNγ induced a higher level of phosphorylated STAT1 and STAT3 than IFNγ alone at 30′, followed by a reduced level of both phospho-STATs at 60′ of stimulation (Figures 5D and 5E). These results suggest that eNAMPT boosts STAT1/3 signaling and accelerates the kinetics of IFNγ-induced STAT1 and 3 phosphorylation. We confirmed this by using a specific STAT3 inhibitor, Stattic (3 μM), and observing the loss of the eNAMPT-mediated boosting effect on IFNγ response is reset (Figure S5C).

To rule out an effect of TLR4 in eNAMPT-induced STATs activation, we analyzed phosphorylation of STAT1 and STAT3 in TLR4-KO PECs. Although we found a faster decay of STAT phosphorylation, the results showed a consistent increase of both phospho-STAT1 and phospho-STAT3 levels after 30′ of treatment (Figures 5F–5G), thus confirming that eNAMPT boosts IFNγ signaling in a TLR4-independent manner.

### eNAMPT is actively secreted during IFNγ-induced M1-polarization

**Figure 6:**
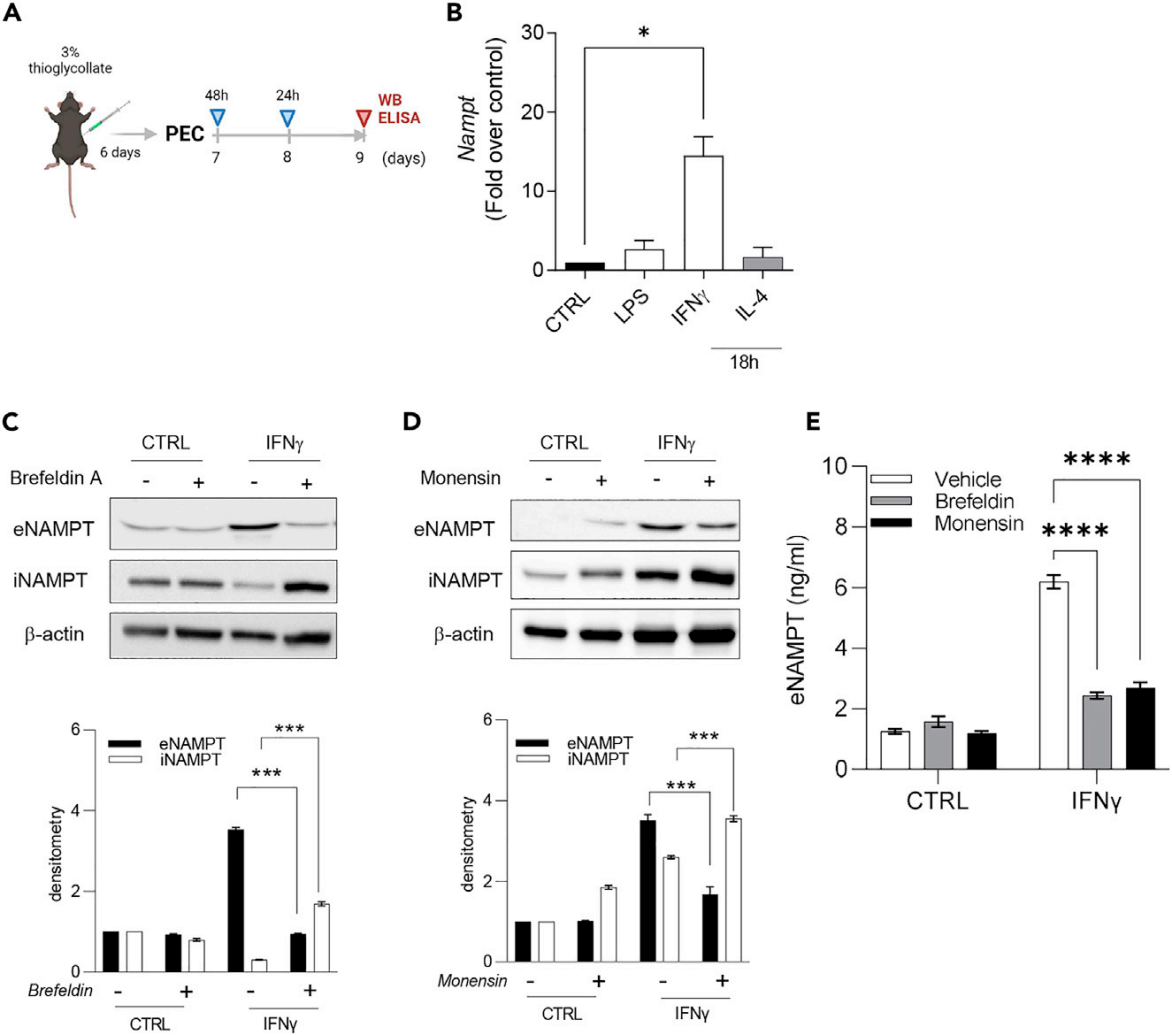
IFNγ upregulates and triggers release of eNAMPT via the canonical pathway (A) Representative scheme of experimental plan (created with BioRender). (B) *Nampt* mRNA levels after IFNγ, LPS, and IL-4 stimulation; mean ± S.E.M. of 4 independent experiments. (C) Representative western blot and densitometry of iNAMPT in total cell lysates and of eNAMPT in medium from PECs treated with IFNγ (200 U/mL) after 48 h in the presence or absence of brefeldin (1 μg/mL) for the last 4 h of incubation. Mean ± S.E.M. of 3 independent experiments. (D) Representative western blot and densitometry of iNAMPT in total cell lysates and of eNAMPT in medium from PECs treated with IFNγ (200 U/mL) after 48 h in the presence or absence of monensin (1 μM) for the last 4 h of incubation. Mean ± S.E.M. of 3 independent experiments; (E) eNAMPT levels evaluated with ELISA of PECs treated with IFNγ (200 U/mL) after 48 h in the presence or absence of monensin (1 μM) or brefeldin (1 μg/mL) the last 4 h of incubation. Mean ± S.E.M. of 3 independent experiments. p value: ∗p < 0.05; ∗∗∗p < 0.001; ∗∗∗∗p < 0.0001.

Our RNAseq analysis shows that the *Nampt* is one of the most IFNγ-upregulated genes (Figure 2D; log2 fold-change of 2.1 over control) and is strikingly further potentiated by the co-stimulation of PECs with eNAMPT (log2 fold-change of 2.75 over control). Accordingly, it has been recently reported that IFNγ upregulates iNAMPT expression in a STAT-dependent manner (Huffaker et al., 2021). We confirmed RNAseq results by qPCR analysis. Despite in THP-1 cells iNAMPT is selectively induced by LPS (Halvorsen et al., 2015), for PECs we observed that *Nampt* transcription is mostly induced by IFNγ (Figure 6B). These results suggest that *Nampt* selectively belongs to the IFNγ signature and prompted us to explore the relationship between IFNγ stimulation and eNAMPT production. First, we evaluated iNAMPT (whole lysates) and eNAMPT (supernatants) levels after 48-h stimulation with IFNγ *via* western blot. As shown in Figures 6C and 6D, densitometric analysis confirmed the upregulation of both intracellular and extracellular forms of NAMPT, upon IFNγ treatment. Importantly, we investigated the mechanism whereby IFNγ induced a consistent and robust release of eNAMPT by treating. IFNγ-activated PECs with brefeldin A (1 μg/mL) or monensin (1 mM). Both inhibitors of the protein transport from ER to Golgi apparatus significantly reduced eNAMPT release (Figures 6C and 6D). To corroborate these findings, we analyzed cell-free supernatants by ELISA, and we obtained superimposable results (Figure 6E). Overall, these results demonstrate that IFNγ triggers PECs to increase eNAMPT production by inducing *Nampt* gene expression and by favoring protein release through the canonical pathway.

## Discussion

eNAMPT is increasingly explored as a drug target in a variety of inflammatory diseases (Colombo et al., 2020; Garcia et al., 2021; Quijada et al., 2021). Being the orchestrators of both initiation and resolution of inflammation, macrophages are pivotal players in many disorders and consequently promising targets for new therapeutic strategies (Sica et al., 2015).

Here, we demonstrate that eNAMPT enhances macrophages-driven inflammation by (1) promoting the chemotactic recruitment of myeloid cells; (2) activating macrophages to express an M1-skewed transcriptional program; and (3) boosting IFNγ-driven transcriptional activation through the potentiation of STAT1/3 phosphorylation.

Macrophages are distributed through the body where they act as crucial gatekeepers of tissue homeostasis and key players of innate and adaptive immune response (Amit et al., 2016). Plasticity is the hallmark of monocytes/macrophages that carry out different responses to the plethora of physiologic and pathologic microenvironmental signals (e.g., microbial products, endogenous alarmins, metabolites, ROS) they are exposed to (Gordon and Mantovani, 2011). Different studies have investigated the effect of eNAMPT on macrophage polarization; however, this issue has remained debated (Travelli et al., 2018). Indeed, looking at the expression of only a few genes, some studies indicate that eNAMPT has an M1 skewing ability (Bermudez et al., 2017; Halvorsen et al., 2015), whereas others show an enhancement of M2-skewed activation. A potential explanation might be found on the complexity of macrophage activation and the source of macrophages used to perform such studies (Murray et al., 2014). For the first time, we have provided a comprehensive transcriptional analysis by RNAseq of primary murine macrophages (PECs) activated by eNAMPT. Therefore, the results allow us to conclusively determine the effect of this protein on macrophage-polarized activation. The description of circulating eNAMPT and its biological activity dates back in time (Fukuhara et al., 2007; Samal et al., 1994), nevertheless its receptor has remained elusive. We have recently shown that this protein may bind and antagonize CCR5 on murine melanoma cells (Torretta et al., 2020); however, in a muscle injury model in zebrafish, the binding of eNAMPT to the CCR5 expressed by muscle stem cells triggers a signaling cascade that supports muscle regeneration (Ratnayake et al., 2021). These studies suggest that eNAMPT might modulate CCR5 activity in a cell-type-dependent manner. Nonetheless, we have observed that eNAMPT-induced M1 PEC activation is not affected by maraviroc, ruling out a contribution of CCR5 for eNAMPT activities in macrophages (not shown).

TLR4 is an alternative receptor of eNAMPT that has emerged by SPR studies and then confirmed by additional evidence (Camp et al., 2015). For example, in human monocytes a TLR4-neutralizing antibody was able to reduce eNAMPT-mediated NF-κB activation (Managò et al., 2019). Here, we investigated the contribution of TLR4 on the effects of eNAMPT by using PECs from TLR4 KO mice. Our results demonstrate that eNAMPT exerts its effects through a TLR4-independent pathway. Given that KEGG analysis points out a consistent enrichment of TLR signaling, it is reasonable to assume that eNAMPT activities are receptor specific and that the receptor could yet belong to the TLR family.

Alongside a receptor interaction, a second line of thought hypothesizes that the enzymatic activity of eNAMPT could be important (Revollo et al., 2007). In the present contribution, we demonstrate that the effect of eNAMPT is superimposable to that of the catalytically inactive NAMPT^H247E^ mutant, thereby ruling out by contribution from metabolism. Therefore, our data re-open the search for the receptors responsible for the actions of eNAMPT.

Strikingly, we also observed that IFNγ induces the expression and release of eNAMPT, thereby providing a positive feedback loop for macrophage-driven inflammation. It is worth noting that eNAMPT has been found to be increased in numerous pathological conditions that are also associated with elevated levels of IFNγ, including autoimmune disorders and sepsis (Chung et al., 2009; Managò et al., 2019; Starr et al., 2017). Our results confirmed the boosting effect of eNAMPT on IFNγ-induced gene expression in human monocyte-derived macrophages, therefore strengthening the potential relevance of eNAMPT neutralization in IFNγ-dependent inflammatory disorders. Accordingly, both we and another group have generated eNAMPT neutralizing antibodies that are able to mitigate inflammation in preclinical models of inflammatory bowel disease, acute lung injury, and ventilatory-induced lung injury (Camp et al., 2015; Colombo et al., 2020; Quijada et al., 2021).

There is abundant literature regarding NAMPT and macrophages. Huffaker et al. have highlighted the role of intracellular NAMPT (iNAMPT) in mediating the effects of IFNγ in tumor-associated macrophages (Huffaker et al., 2021). Such observations prevented us from using specific inhibitors of this enzyme, as they would have had a confounding effect. Audrito et al., showed that monocytes from leukemic patients stimulated with eNAMPT increase their M2-phenotype (Audrito et al., 2015). Last, Li et al. showed an effect of eNAMPT on STAT3 phosphorylation (Li et al., 2008). Our manuscript complements these observations and shows for the first time that, in unskewed macrophages, eNAMPT induces a M1 phenotype and strongly synergizes with IFNγ.

In conclusion, we have demonstrated that eNAMPT promotes inflammation by favoring both the recruitment of myeloid cells and the induction of an inflammatory transcriptional program. Moreover, IFNγ triggers macrophages to upregulate and release eNAMPT that boosts IFNγ-driven transcriptional activation, thereby suggesting eNAMPT as a new amplifier of the cytokine storm.

### Limitation of the study

The main limitation of the study is given by the fact that although it presents solid data excluding the involvement of TLR4 and of the enzymatic activity, the responsible receptor remains unknown. Other limitations may be as follows: (1) the high concentration of eNAMPT that does not reflect the amount of the cytokine in the inflammatory milieu, but we used amounts that are coherent with the literature; (2) we did not investigate all the possible pathways that may be activated by eNAMPT, but they will be one of our interests in the future.

## STAR★Methods

### Key resources table

**Table:** 

REAGENT or RESOURCE	SOURCE	IDENTIFIER
**Antibodies**		

BD Horizon^TM^ Rat anti-mouse CD45 Clone 30-F11	BD Bioscience	Cat #564279 RRID: AB_2651134
BD Horizon^TM^ Armenian hamster anti-mouse CD3 Clone 145-2C11	BD Bioscience	Cat #566494 RRID: AB_2744393
BD Pharmingen^TM^ Rat anti-mouse Ly6C Clone AL-21	BD Bioscience	Cat #553104 RRID: AB_394628
BD Optibuild^TM^ Rat anti-mouse Ly6G Clone 1A8	BD Bioscience	Cat #740157 RRID: AB_2739910
BD Optibuild^TM^ Rat anti-mouse F4/80 Clone 6F12	BD Bioscience	Cat #744339 RRID: AB_2742166
BD Horizon^TM^ Rat anti-mouse CD86 Clone GL1	BD Bioscience	Cat #560450 RRID: AB_1645280
BD Pharmingen^TM^ Rat anti-mouse CD206 Clone MR5D3	BD Bioscience	Cat #565250 RRID: AB_2739133
BD Optibuild^TM^ Rat anti-mouse CD/11b Clone M1/70	BD Bioscience	Cat #550282 RRID: AB_393577
BD Horizon^TM^ Fixable viability stain Live/Dead	BD Bioscience	Cat #564997 RRID: AB_2869637
Anti-NAMPT	Adipogen	Cat#ALX-804-717-C050 RRID: AB_11180657
Anti-NAMPT	Genetex	Cat #GTX128973 RRID: AB_2810933
Anti-b-actin	Sigma	Cat #A1978 RRID: AB_476692
Rb anti-phospho-STAT3 (Tyr705) (D3A7)	Cell Signaling	Cat #9145 RRID: AB_2491009
mo anti-STAT3 (124H6)	Cell Signaling	Cat #9139 RRID: AB_331757
Rb anti-phospho-STAT1 (Tyr701) (D4A7)	Cell Signaling	Cat #7649 RRID: AB_10950970
Rb anti-STAT1 (D1K9Y)	Cell Signaling	Cat #14994 RRID: AB_2737027

**Bacterial and virus strains**		

ClearColi BL21(DE3)	Lucigen	Cat #60810-1

**Chemicals, peptides, and recombinant proteins**		

Thioglycollate	BD Bioscience	Cat #L007454
RPMI medium	Merck Life Science	Cat #R650
FBS	Gibco	Cat #A4766801
Penicillin/Streptomycin	Merck Life Science	Cat #P4333
Glutamine	Merck Life Science	Cat #G6784
LPS *Escherichia coli* O111:B4	Merck Life Science	Cat #L2630
Histopaque-1191	Merck Life Science	Cat #11191
Histopaque-10771	Merck Life Science	Cat #10771
hM-CSF	Peprotech	Cat #300-25
hNAMPT	Peprotech	Cat #130-09
hIFNg	Peprotech	Cat #300-02
mIFNg	Peprotech	Cat #315-05
mIL-4	Peprotech	Cat #214-14
mIL-6	Peprotech	Cat #216-16
mGM-CSF	Peprotech	Cat #315-03
mIL-1b	Peprotech	Cat #211-11B
Bradford Protein Assay	Merck Life Science	Cat #B6916
ECL	Thermo Scientific	Cat #32106

**Critical commercial assays**		

ToxinSensor Chromogenic LAL Endotoxin Assay kit	Genescript	Cat #L00350
Murine NAMPT ELISA KIT	Adipogen	Cat#AG-45A-0007YEK-KI01
Human NAMPT ELISA KIT	Adipogen	Cat#AG-45A-0006YEK-KI01
SENSIFAST kit	Bioline/Aurogene	Cat #BIO-65054
Deposited data		
RNAseq data	GEO	GSE189104

**Experimental models: Organisms/strains**		

C57BL/6	Envigo	RRID: MGI:5658455

**Oligonucleotides**		

Primer for qPCR in Table S1	Table S1	N/A

**Software and algorithms**		

GraphPad Prism V9	GraphPad	RRID: SCR_002798
FlowJo	FlowJo	RRID: SCR_008520
BD FACSDiva 8.0.2	BD Bioscience	N/A

### Resource availability

#### Lead contact

Further information and requests for resources and reagents should be directed to and will be fulfilled by the lead contact, Armando A. Genazzani (armando.genazzani@uniupo.it).

#### Materials availability

This study did not generate new materials or reagents.

#### Data and code availability

All data generated or analysed during this study are available upon request. The RNA-seq data have been deposited in the Gene Expression Omnibus (GEO) database under the accession GSE189104. This paper does not report original code. Any additional required to reanalyse the data reported in this paper is available from the lead contact upon request.

### Experimental model and subject details

#### Isolation of murine peritoneal macrophages

Animal care was in compliance with Italian regulations on protection of animals used for experimental purposes and were authorized by the Ministry of Health (120/2018 DB064.27 of 04/10/2017 and 983/2020-PR DB064.62 of 14/10/2020). C57BL/6 (WT or TLR4-KO, Jackson Laboratory) male 8-weeks-old mice were injected in the peritoneal cavity with 1 mL of 3% Brewer thioglycollate medium (BD Bioscience). After 5 days, the mice were euthanized. After retracting the abdominal skin, exposing the peritoneal wall, 5 mL of sterile PBS were injected closed to abdominal adipose tissue. The liquid in the peritoneal cavity was shacked, aspirated with the syringe closed to sternum and collected for macrophage purification.

2 or 3 × 10ˆ6 cells peritoneal exudate cells (PECs) were seeded in RPMI-FBS free Medium (RPMI, with 10 U/mL Penicillin, 100 μg/mL streptomycin and 1% L-glutamine, Merck Life Science) and left 1 h in incubator at 37°C 5% CO_2_. Next, the non-macrophage cells were vigorously washed away with PBS and culture in complete RPMI-medium (RPMI with addition of 10% of FBS, Gibco, Thermo Fisher Scientific) at 37°C 5%CO_2_ for at least 1 h. Macrophages were treated as following described.

#### Air pouch

Ten-week-old C57Bl/6 (WT and TLR4-KO) mice were used, and all experiments were performed under isoflurane anaesthesia. Mice were subcutaneously injected with 3 mL of sterile air on the dorsal region, at days 0 and 3. At day 6, 500 ng/mL of eNAMPT or 100 ng/mL of LPS were injected in the pouches. Control mice were administered with PBS. After 6 h, the cells recruited in the pouches were harvested with PBS, stained and analysed by flow cytometry.

#### Human monocyte-derived macrophages (MDM)

All healthy volunteers gave written, informed consent to blood collection and the procedure was approved by the local institutional review board (protocol 583/CE). Peripheral blood (30 mL) was drawn and anticoagulated with 0.32% w/v sodium citrate. PMBCs were isolated through a gradient formation using Histopaque-11191 and Histopaque-10771 (Sigma-Aldrich). PBMCs were washed once with PBS, pelleted at 1500 × g for 10′ and re-suspended in complete RPMI. The cells were seeded in a 6-multiwell plate and incubated for 2 h. Then, the non-adherent cells were removed by PBS with calcium and magnesium, the remaining monocytes were cultured in complete RPMI with 10 ng/mL of M-CSF. After 3 days, the medium was replaced with fresh M-CSF-added medium. After 6 days, monocyte-derived macrophages (MDM) were ready to be treated for the experiments.

### Method details

#### RNA sequencing and data analysis

Libraries were generated from total RNA (5 samples/conditions) of PECs treated with recombinant murine NAMPT (500 ng/mL) or murine IFNγ (Peprotech, 200 U/mL) for 4 h. RNA was extracted using SPLIT RNA Extraction Kit (Lexogen, Vienna, Austria). Total RNA quality was evaluated using the Agilent 2100 Bioanalyzer System.

RNA samples were processed using the QuantSeq 3′ mRNA-Seq Library Prep Kit (Lexogen, Vienna, Austria) and sequenced on an Illumina NextSeq 500. Read counts were normalized for effective library size, and differentially expressed genes (DEGs) were analysed using DESeq2.21 DEGs were identified by a FDR <0.05 and an absolute fold change >1.

The functional analysis of the identified differentially expressed genes was performed with DAVID v6.8 and Panther Classification System v12.0 by uploading all the DEGs. PPI were created using STRING v10.5 by uploading all the DEGs and only connected proteins were considered to build the network map. Venn diagrams were designed using Venny free on-line tool (http://bioinfogp.cnb.csic.es/tools/venny/) to picture intersections between class comparison results and to select the genes of interest.

Next, mRNA accession numbers of DEGs were subjected to TF binding motif enrichment analysis using enriched groups of −950 base pair sequence to +50 base pair using Pscan (Zambelli et al., 2009) and the JASPAR database.

#### Recombinant murine eNAMPT purification

Wild-type murine full-length NAMPT (ORF GenBank BC018358) and NAMPT^H247E^ (obtained by mutagenesis with QuikChange XL II kit, Agilent Stratagenewas) were cloned in pET28a (NdeI/EcoRI) and expressed in ClearColi BL21(D3) (induction with IPTG 0.5 mM for 3 h at 21°C) and purified by His-tag affinity chromatography with NiNTA Superflow resin (Qiagen). Endotoxin levels were assessed with ToxinSensor Chromogenic LAL Endotoxin Assay kit (GeneScript). Only preparations with less than 0.1 EU/mL endotoxin levels were utilized. NAMPT and NAMPT^H247E^ activity was tested accordingly (Amici et al., 2017).

#### Measurement of eNAMPT levels in cell medium

For eNAMPT measurement, 3 × 10ˆ6 cells were seeded in 6-well plates and cultured in serum-free conditions, with or without treatments, for 48 h. Then, the conditioned medium was collected and 50μL were analysed by Western blotting. Experiments were performed in serum-free conditions to avoid aspecific immunoglobulin signals and because of the possible presence of eNAMPT in FBS. In parallel, some no-starved samples were analysed for eNAMPT concentrations using a commercially available sandwich enzyme-linked immunosorbent assay for human or murine NAMPT (ELISA kit from AdipoGen Inc, Seoul Korea).

#### Gene expression analysis

Cells were lysed with Trizol reagent (Life-technologies) and RNA was extracted with chloroform. 1 μg RNA was reverse transcripted with SENSIFAST kit as manufacturer’s protocol (Aurogene) and 20 ng of cDNA were used to perform qPCR with SYBR-green (Bio-Rad) and CFX96 Real-Time System (Bio-Rad). Gene expression results were normalized to actin as housekeeping gene. The sequences of gene-specific primers are reported in Table S1.

#### Wound-healing assay

3 × 10ˆ6 cells were seeded in 6-well plates. We performed a cross-shaped scratch with a tip. Then, the cells were washed twice with PBS to remove residual cell debris. Cells were treated with eNAMPT (500 ng/mL) and fMLP (1 μM) and wound closure was monitored up to 48 h. Pictures were taken at different time points by Leica DM IL LED (Leica Microsystem) and areas were analysed using Image J software (National Institutes of Health, MD, USA).

#### Transwell migration assay

3 × 10^4^ cells were seeded on the top of 12 μm Transwell inserts and the lower chamber was filled up with media containing 10% foetal bovine serum, in presence or not with eNAMPT (500 ng/mL) and fMLP (1 μM). After 24 h, the migrating cells were fixed using methanol and stained with 0.1% crystal violet. Images were captured by Leica DM IL LED (Leica Microsystem) and cells were counted.

#### Flow cytometry analysis

Cells were stained in 0.5% FBS and 1 mM EDTA in HBSS solution with the antibodies reported in Table S2. Cells were acquired using BD Symphony™, and data were analysed using BD FACSDiva 8.0.2 and FlowJo (10.6.1) software.

#### Treatments

C269 (10 μg/mL), control IgG1 (10 μg/mL) and recombinant murine NAMPT (rNAMPT, 500 ng/mL) were produced and purified as previously described (Colombo et al., 2020). PECs were treated with LPS (100 ng/mL lipopolysaccharides from *Escherichia coli* O 111:B4, Sigma, Cat. No. L2630), murine IFNγ (Peprotech, 200 U/mL), murine IL-4 (Peprotech, 20 ng/mL), murine IL-6 (100 ng/mL), murine GM-CSF (50 ng/mL) and murine IL-1β (50 ng/mL). Stattic (Merck Life Science) was used at 3μM for 1 h.

For treatment with C269, a 6-multiwell plate was coated with the antibodies in a 100 mM of sodium bicarbonate solution O.N. After that, the plate was washed and incubated with medium containing eNAMPT at 37°C for 1 h. Then 0 ,4 μm Transwell Inserts seeded with PECs were added to the plate.

MDM were treated with human IFNγ (Peprotech, 200 U/mL) and/or murine NAMPT (Peprotech, 500 ng/mL).

#### Western Blot analysis

PECs were lysed in RIPA Buffer (20 mM HEPES, 100 mM NaCl, 5 mM EDTA, 1% Nonidet-P40+ Protease & Phosphatase Inhibitor Cocktail, Merck Life Science). Proteins were quantified by Bradford Protein Assay (Merck Life Science) and 30 μg of proteins were resolved on SDS-PAGE, transferred on nitrocellulose membrane by the TurboBlot system (BioRad, Hemel Hempstead, UK). Proteins were detected with primary antibodies and peroxidase-conjugated secondary antibodies (Bio-Rad) and resolved by chemiluminescence analysis using ECL (Thermo Fisher Scientific). Densitometry analysis was performed with the Image Lab program (Bio-Rad, Hemel Hempstead, UK). The list of primary antibodies used are listed under “Reagents.”

#### Reagents

Antibodies used were as follows: mouse (Mo) anti-NAMPT from AdipoGen (OMNI379); rabbit (Rb) anti-NAMPT GTX128973 from GeneTex; Mo anti-βactin A1978 from Sigma, Rb anti-phospho-STAT3 (Tyr705) (D3A7) from Cell Signaling, mo anti-STAT3 (124H6) from Cell Signaling, Rb anti-phospho-STAT1 (Tyr701) (D4A7) from Cell Signaling and Rb anti-STAT1 (D1K9Y) from Cell Signaling.

### Quantification and statistical analysis

Data are presented as mean ± SEM. The normality of data distributions was evaluated using the Shapiro-Wilk test. Parametric (unpaired t-test and One-way analysis of variance (ANOVA) followed by Tukey’s post-hoc) or non-parametric (Mann-Whitney U test and One-way Kruskal-Wallis H test followed by Dunn’s post-hoc) statistical analysis were used. All statistical assessments were two-sided and a value of p < 0.05 was considered statistically significant. Statistical analysis was performed using GraphPad Prism software (GraphPad Software, Inc., USA).

## Supplemental information

Document S1. Figures S1–S5 and Tables S1 and S2

Table S3. Combinatory evaluation, related to Figure 4 Evaluation of possible additivity or synergy of IFNγ+eNAMPT between the most up-regulated genes revealed by RNAseq analysis.

## References

[bib1] Adams D.O., Hamilton T.A. (1984). The cell biology of macrophage activation. Annu. Rev. Immunol..

[bib42] Amici A., Grolla A.A., Del Grosso E., Bellini R., Bianchi M., Travelli C., Garavaglia S., Sorci L., Raffaelli N., Ruggieri S. (2017). Synthesis and Degradation of Adenosine 5’-Tetraphosphate by Nicotinamide and Nicotinate Phosphoribosyltransferases. Cell Chem. Biol..

[bib2] Amit I., Winter D.R., Jung S. (2016). The role of the local environment and epigenetics in shaping macrophage identity and their effect on tissue homeostasis. Nat. Immunol..

[bib3] Audrito V., Serra S., Brusa D., Mazzola F., Arruga F., Vaisitti T., Coscia M., Maffei R., Rossi D., Wang T. (2015). Extracellular nicotinamide phosphoribosyltransferase (NAMPT) promotes M2 macrophage polarization in chronic lymphocytic leukemia. Blood.

[bib4] Bermudez B., Dahl T.B., Medina I., Groeneweg M., Holm S., Montserrat-de la Paz S., Rousch M., Otten J., Herias V., Varela L.M. (2017). Leukocyte overexpression of intracellular NAMPT attenuates atherosclerosis by regulating PPARγ-dependent monocyte differentiation and function. Arterioscler Thromb. Vasc. Biol..

[bib5] Camp S.M., Ceco E., Evenoski C.L., Danilov S.M., Zhou T., Chiang E.T., Moreno-Vinasco L., Mapes B., Zhao J., Gursoy G. (2015). Unique toll-like receptor 4 activation by NAMPT/PBEF induces NFκB signalling and inflammatory lung injury. Sci. Rep..

[bib6] Chung C.P., Long A.G., Solus J.F., Rho Y.H., Oeser A., Raggi P., Stein C.M. (2009). Adipocytokines in systemic lupus erythematosus: relationship to inflammation, insulin resistance and coronary atherosclerosis. Lupus.

[bib7] Colombo G., Clemente N., Zito A., Bracci C., Colombo F.S., Sangaletti S., Jachetti E., Ribaldone D.G., Caviglia G.P., Pastorelli L. (2020). Neutralization of extracellular NAMPT (nicotinamide phosphoribosyltransferase) ameliorates experimental murine colitis. J. Mol. Med.

[bib8] Curat C.A., Wegner V., Sengenès C., Miranville A., Tonus C., Busse R., Bouloumié A. (2006). Macrophages in human visceral adipose tissue: increased accumulation in obesity and a source of resistin and visfatin. Diabetologia.

[bib9] Dahl T.B., Yndestad A., Skjelland M., Øie E., Dahl A., Michelsen A., Damås J.K., Tunheim S.H., Ueland T., Smith C. (2007). Increased expression of visfatin in macrophages of human unstable carotid and coronary atherosclerosis: possible role in inflammation and plaque destabilization. Circulation.

[bib10] Das A., Yang C.-S., Arifuzzaman S., Kim S., Kim S.Y., Jung K.H., Lee Y.S., Chai Y.G. (2018). High-resolution mapping and dynamics of the transcriptome, transcription factors, and transcription Co-factor networks in classically and alternatively activated macrophages. Front. Immunol..

[bib11] Fukuhara A., Matsuda M., Nishizawa M., Segawa K., Tanaka M., Kishimoto K., Matsuki Y., Murakami M., Ichisaka T., Murakami H. (2007). Retraction. Science.

[bib12] Garcia A.N., Casanova N.G., Valera D.G., Sun X., Song J.H., Kempf C.L., Moreno-Vinasco L., Burns K., Bermudez T., Valdez M. (2021). Involvement of eNAMPT/TLR4 signalling in murine radiation pneumonitis: protection by eNAMPT neutralization. Transl Res..

[bib13] Garten A., Schuster S., Penke M., Gorski T., de Giorgis T., Kiess W. (2015). Physiological and pathophysiological roles of NAMPT and NAD metabolism. Nat. Rev. Endocrinol..

[bib14] Gordon S., Mantovani A. (2011). Diversity and plasticity of mononuclear phagocytes. Eur. J. Immunol..

[bib15] Grolla A.A., Torretta S., Gnemmi I., Amoruso A., Orsomando G., Gatti M., Caldarelli A., Lim D., Penengo L., Brunelleschi S. (2015). Nicotinamide phosphoribosyltransferase (NAMPT/PBEF/visfatin) is a tumoural cytokine released from melanoma. Pigment Cell Melanoma Res.

[bib16] Halvorsen B., Espeland M.Z., Andersen G.Ø., Yndestad A., Sagen E.L., Rashidi A., Knudsen E.C., Skjelland M., Skagen K.R., Krohg-Sørensen K. (2015). Increased expression of NAMPT in PBMC from patients with acute coronary syndrome and in inflammatory M1 macrophages. Atherosclerosis.

[bib17] Huffaker T.B., Ekiz H.A., Barba C., Lee S.-H., Runtsch M.C., Nelson M.C., Bauer K.M., Tang W.W., Mosbruger T.L., Cox J.E. (2021). A Stat1 bound enhancer promotes Nampt expression and function within tumor associated macrophages. Nat. Commun..

[bib18] Laudes M., Oberhauser F., Schulte D.M., Freude S., Bilkovski R., Mauer J., Rappl G., Abken H., Hahn M., Schulz O. (2010). Visfatin/PBEF/Nampt and resistin expressions in circulating blood monocytes are differentially related to obesity and type 2 diabetes in humans. Horm. Metab. Res..

[bib19] Li Y., Zhang Y., Dorweiler B., Cui D., Wang T., Woo C.W., Brunkan C.S., Wolberger C., Imai S., Tabas I. (2008). Extracellular Nampt promotes macrophage survival via a nonenzymatic interleukin-6/STAT3 signalling mechanism. J. Biol. Chem..

[bib20] Lu Q., Yuan K., Li X., Jiang H., Huo G., Jia W., Huang G., Xu A. (2020). Detecting migration and infiltration of neutrophils in mice. J. Vis. Exp..

[bib21] Managò A., Audrito V., Mazzola F., Sorci L., Gaudino F., Gizzi K., Vitale N., Incarnato D., Minazzato G., Ianniello A. (2019). Extracellular nicotinate phosphoribosyltransferase binds Toll like receptor 4 and mediates inflammation. Nat. Commun..

[bib22] Moschen A.R., Kaser A., Enrich B., Mosheimer B., Theurl M., Niederegger H., Tilg H. (2007). Visfatin, an adipocytokine with proinflammatory and immunomodulating properties. J. Immunol..

[bib23] Murray P.J., Allen J.E., Biswas S.K., Fisher E.A., Gilroy D.W., Goerdt S., Gordon S., Hamilton J.A., Ivashkiv L.B., Lawrence T. (2014). Macrophage activation and polarization: nomenclature and experimental guidelines. Immunity.

[bib24] Ortiz-Masiá D., Hernández C., Quintana E., Velázquez M., Cebrián S., Riaño A., Calatayud S., Esplugues J.V., Barrachina M.D. (2010). iNOS-derived nitric oxide mediates the increase in TFF2 expression associated with gastric damage: role of HIF-1. FASEB J..

[bib25] Piccolo V., Curina A., Genua M., Ghisletti S., Simonatto M., Sabò A., Amati B., Ostuni R., Natoli G. (2017). Opposing macrophage polarization programs show extensive epigenomic and transcriptional cross-talk. Nat. Immunol..

[bib26] Qing Y., Stark G.R. (2004). Alternative activation of STAT1 and STAT3 in response to interferon-gamma. J. Biol. Chem..

[bib27] Quijada H., Bermudez T., Kempf C.L., Valera D.G., Garcia A.N., Camp S.M., Song J.H., Franco E., Burt J.K., Sun B. (2021). Endothelial eNAMPT amplifies pre-clinical acute lung injury: efficacy of an eNAMPT-neutralising monoclonal antibody. Eur. Respir. J..

[bib28] Ratnayake D., Nguyen P.D., Rossello F.J., Wimmer V.C., Tan J.L., Galvis L.A., Julier Z., Wood A.J., Boudier T., Isiaku A.I. (2021). Macrophages provide a transient muscle stem cell niche via NAMPT secretion. Nature.

[bib29] Revollo J.R., Körner A., Mills K.F., Satoh A., Wang T., Garten A., Dasgupta B., Sasaki Y., Wolberger C., Townsend R.R. (2007). Nampt/PBEF/Visfatin regulates insulin secretion in beta cells as a systemic NAD biosynthetic enzyme. Cell Metab..

[bib30] Samal B., Sun Y., Stearns G., Xie C., Suggs S., McNiece I. (1994). Cloning and characterization of the cDNA encoding a novel human pre-B-cell colony-enhancing factor. Mol. Cell. Biol..

[bib31] Sica A., Erreni M., Allavena P., Porta C. (2015). Macrophage polarization in pathology. Cell Mol. Life Sci..

[bib32] Starr A.E., Deeke S.A., Ning Z., Chiang C.-K., Zhang X., Mottawea W., Singleton R., Benchimol E.I., Wen M., Mack D.R. (2017). Proteomic analysis of ascending colon biopsies from a paediatric inflammatory bowel disease inception cohort identifies protein biomarkers that differentiate Crohn’s disease from UC. Gut.

[bib33] Tanaka M., Nozaki M., Fukuhara A., Segawa K., Aoki N., Matsuda M., Komuro R., Shimomura I. (2007). Visfatin is released from 3T3-L1 adipocytes via a non-classical pathway. Biochem. Biophys. Res. Commun..

[bib34] Torretta S., Colombo G., Travelli C., Boumya S., Lim D., Genazzani A.A., Grolla A.A. (2020). The cytokine nicotinamide phosphoribosyltransferase (eNAMPT; PBEF; visfatin) acts as a natural antagonist of C-C Chemokine receptor type 5 (CCR5). Cells.

[bib35] Travelli C., Colombo G., Mola S., Genazzani A.A., Porta C. (2018). NAMPT: a pleiotropic modulator of monocytes and macrophages. Pharmacol. Res..

[bib36] Van den Bergh R., Morin S., Sass H.J., Grzesiek S., Vekemans M., Florence E., Thanh Thi Tran H., Imiru R.G., Heyndrickx L., Vanham G. (2012). Monocytes contribute to differential immune pressure on R5 versus X4 HIV through the adipocytokine visfatin/NAMPT. PLoS One.

[bib41] Wang T., Zhang X., Bheda P., Revollo J.R., Imai S., Wolberger C. (2006). Structure of Nampt/PBEF/visfatin, a mammalian NAD+ biosynthetic enzyme. Nat. Struct. Mol. Biol..

[bib37] Wu X.-T., Yang Z., Ansari A.R., Xiao K., Pang X.-X., Luo Y., Song H. (2018). Visfatin regulates the production of lipopolysaccharide-induced inflammatory cytokines through p38 signalling in murine macrophages. Microb. Pathog..

[bib38] Yoshida M., Satoh A., Lin J.B., Mills K.F., Sasaki Y., Rensing N., Wong M., Apte R.S., Imai S.-I. (2019). Extracellular vesicle-contained eNAMPT delays aging and extends lifespan in mice. Cell Metab..

[bib39] Yun M.R., Seo J.M., Park H.Y. (2014). Visfatin contributes to the differentiation of monocytes into macrophages through the differential regulation of inflammatory cytokines in THP-1 cells. Cell Signal.

[bib40] Zambelli F., Pesole G., Pavesi G. (2009). Pscan: finding over-represented transcription factor binding site motifs in sequences from co-regulated or co-expressed genes. Nucleic Acids Res..

