# Supplementary material for: Extracellular nicotinamide phosphoribosyltransferase boosts IFNγ-induced macrophage polarization independently of TLR4
Source: iScience. 2022 Mar 23;25(4):104147. doi: 10.1016/j.isci.2022.104147 (PMC8990213; doi:10.1016/j.isci.2022.104147)
Supplement: Document S1. Figures S1–S5 and Tables S1 and S2 [file mmc1.pdf]

iScience, Volume *25*

Supplemental information

Extracellular nicotinamide phosphoribosyltransferase boosts IFNγ-induced macrophage polarization independently of TLR4

Giorgia Colombo, Cristina Travelli, Chiara Porta, and Armando A. Genazzani

Supplementary Table I, primers used for qPCR, related to STAR Methods

Gene	Forward Primer (5'-> 3')	Reverse Primer (5'->3')
Mm_B-Actin	ATGACCCAAGCCGAGAAGG	CGGCCAAGTCTTAGAGTTGTTG
Mm_Tnf	AGTTCTATGGCCCAGACCCTC	CAGGCTTGTCACTCGAATTTTG
Mm_Nampt	GCAGAAGCCGAGTTCAACATC	TTTTCACGGCATTCAAAGTAGGA
Mm_Il6	TGTTCTCTGGGAAATCGTGGA	AAGTGCATCATCGTTGTTCATACA
Mm_Cox2	GACCGCAATGAACTTCGGGA	TCCATTAGGTCTCTAAAGCCGAG
Mm_Il1	GATGAGGACATGAGCACCTTCTT	GCAGGTTATCATCATCATCCCA
Mm_Il18	GACTCTTGCGTCAACTTCAAGG	CAGGCTGTCTTTTGTCAACGA
Mm_Il23a	ATGCTGGATTGCAGAGCAGTA	ACGGGGCACATTATTTTTAGTCT
Mm_Il12b	TGGTTTGCCATCGTTTTGCTG	ACAGGTGAGGTTCACTGTTTCT
Mm_Ifnb	CAGCTCCAAGAAAGGACGAAC	GGCAGTGTAACTCTTCTGCAT
Mm_Nos2	CGAAACGCTTCACTTCCAA	TGAGCCTATATTGCTGTGGCT
Mm_Cxcl9	GGAGTTCGAGGAACCCTAGTG	GGGATTTGTAGTGGATCGTGC
Mm_Cxcl10	CCAAGTGCTGCCGTCATTTTC	GGCTCGCAGGGATGATTTCAA
Mm_Ccl17	TACCATGAGGTCACTTCAGATGC	GCACTCTCGGCCTACATTGG
Mm_ArgI	AACACGGCAGTGGCTTTAACC	GGTTTTCATGTGGCGCATTC
Mm_Ym1	TCACAGGTCTGGCAATTCTTCTG	TTTGTCCTTAGGAGGGCTTCCTCG
Mm_Fizz1	GGTCCCAGTGCATATGGATGAGACCATA GA	CACCTCTTCACTCGAGGGACAGTTGGCAGC
Mm_Irf1	AACCAAATCCCAGGGCTGAT	GGAACAGACAGGCATCCTTG
Mm_Ccl7	CATGCTGCTATGTCAAGAAACA	TAACAGCTTCCCAGGGACAC
Mm_Ccl8	AGTCACCTGCTGCTTTCATG	TGGGGCACTGGATATTGTTG
Mm_Ccl12	GCTACCACCATCAGTCCTCA	GCTTCCGGACGTGAATCTTC
Mm_Ccr2	GCCTGATCCTGCCTCTACTT	CAAAGATGAGCCTCACAGCC
Mm_Cxcl1	CCAGAGCTTGAAGGTGTTGC	TCTGAACCAAGGGAGCTTCA
Mm_Cxcl5	AGGAGGTCTGTCTGGATCCA	CACTGGCCGTTCTTTCCAC
Mm_IcosL	TGCCTTACAAGTCTCCAGGG	GGCATGTGAACTCCTGGGTA

Mm_Myd88	CAGAACCAGGAGTCCGAGAA	AAGGCATCGAAAAGTTCCGG
Mm_Nod1	TTCAATGGCATCTCTCCGGA	AGCGTCTGGTTCACTCTCAG
Mm_Notch2	TGTGAGCGGAATATCGACGA	GACAGCGGCAATTGTAGGTA
h_Il1	CCTACTCACTTAAAGCCCGCCT	TTAGAACCAAATGTGGCCGTG
h_Il12b	GACATTCTGCGTTCAGGTCCAG	CATTTTTGCGGCAGATGACCGTG
h_Tnf	ACGAACATCCAACCTTCCCA	CCCAATTCTCTTTTTGAGCCAG
h_Il10	TTAAGGGTTACCTGGGTTGCCAAGC	TCTTGGTTCTCAGCTTGGGGCATCA
h_ArgI	CTGACTGGAGAGCTCAAGTGCA	TCGTGGCTGTCCCTTTGAGAA
h_Il6	CCAGCTATGAACTCCTTCTC	GCTTGTTCCTCACATCTCTC
h_Cxcl9	CCAGTAGTGAGAAAGGGTCGC	AGGGCTTGGGGCAAATTGTT
h_Cxcl10	GTGGCATTCAAGGAGTACCTC	TGATGGCCTTCGATTCTGGATT

Supplementary Table II, antibodies used for Flow Cytometry, related to STAR Methods

**Marker**	**Fluorophore**	**Brand**
CD45	BUV395	BD Horizon™ Rat anti-mouse
CD3	BB700	BD Horizon™ Armenian hamster anti-mouse
Ly6C	BV786	BD Pharmingen™ Rat anti-mouse
Ly6G	BV480	BD Optibuild™ Rat anti-mouse
F4/80	BUV661	BD Optibuild™ Rat anti-mouse
CD86	BV650	BD Horizon™ Rat anti-mouse
CD206	AF647	BD Pharmingen™ Rat anti-mouse
CD11b	BUV563	BD Optibuild™ Rat anti-mouse
LIVE/DEAD	APC-H7	BD Horizon™ Fixable viability stain

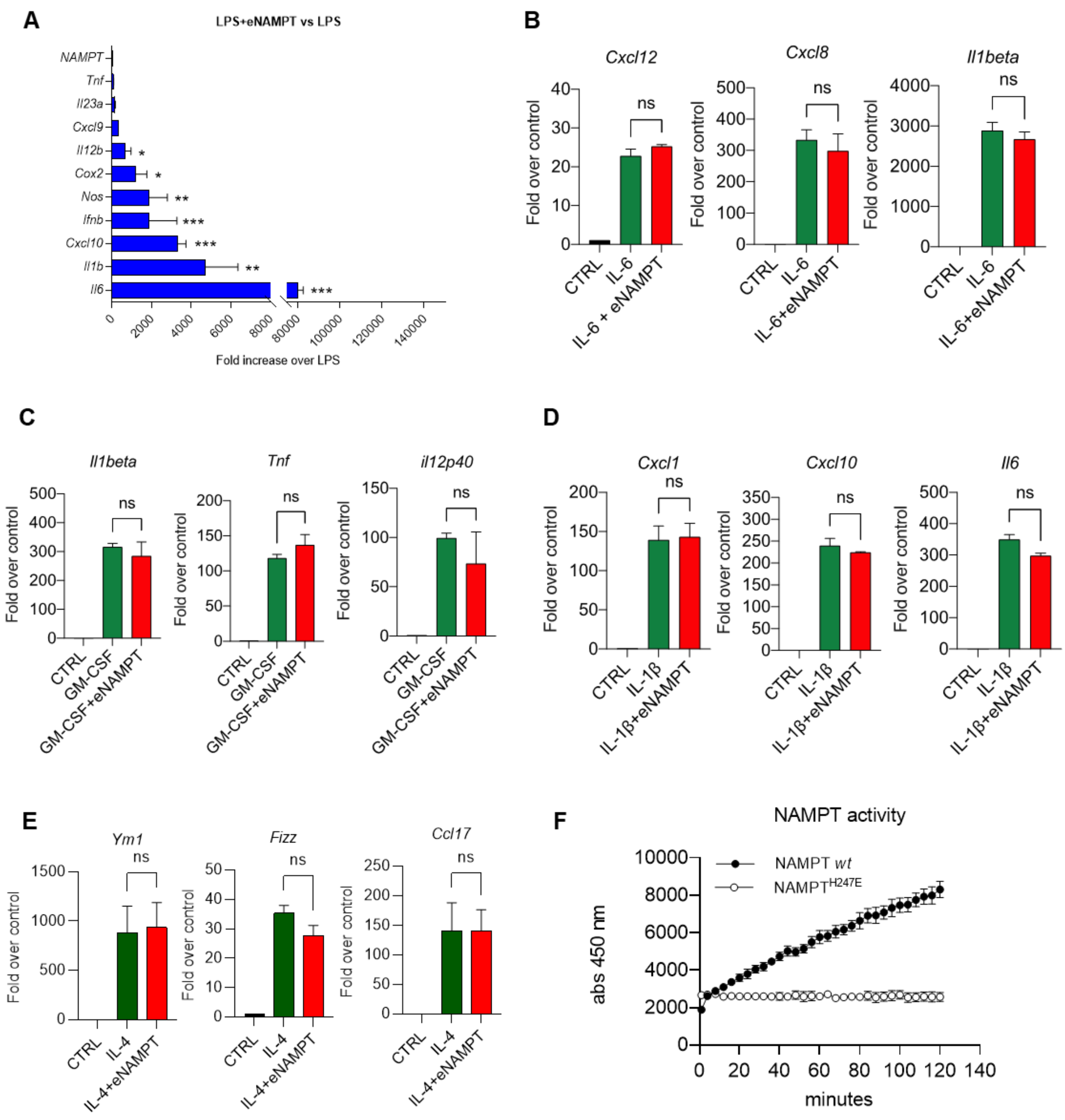

Supplementary Figure 1 related to Figure 1, eNAMPT does not synergize with other cytokines. (**A**) Gene expression changes of the indicated genes in response to eNAMPT (500 ng/ml) and LPS (100ng/ml) in murine PECs. Mean + S.E.M. of 7 independent experiments. (**B-D**) Fold changes of IL-6 (100ng/ml), GM-CSF (50ng/ml), IL-1β- (50ng/ml) dependent genes in PECs treated in the presence or absence with eNAMPT (500 ng/ml) for 4 hours. Mean ± S.E.M. of 3 independent experiments. (**E**) Fold changes of IL-4-dependent genes in PECs treated in the presence or absence with eNAMPT (500 ng/ml) for 18 hours. Mean ± S.E.M. of 3 independent experiments. (**F**) *In vitro* NAMPT and NAMPT^{H247E} enzymatic activity determination, n=2 independent experiments.

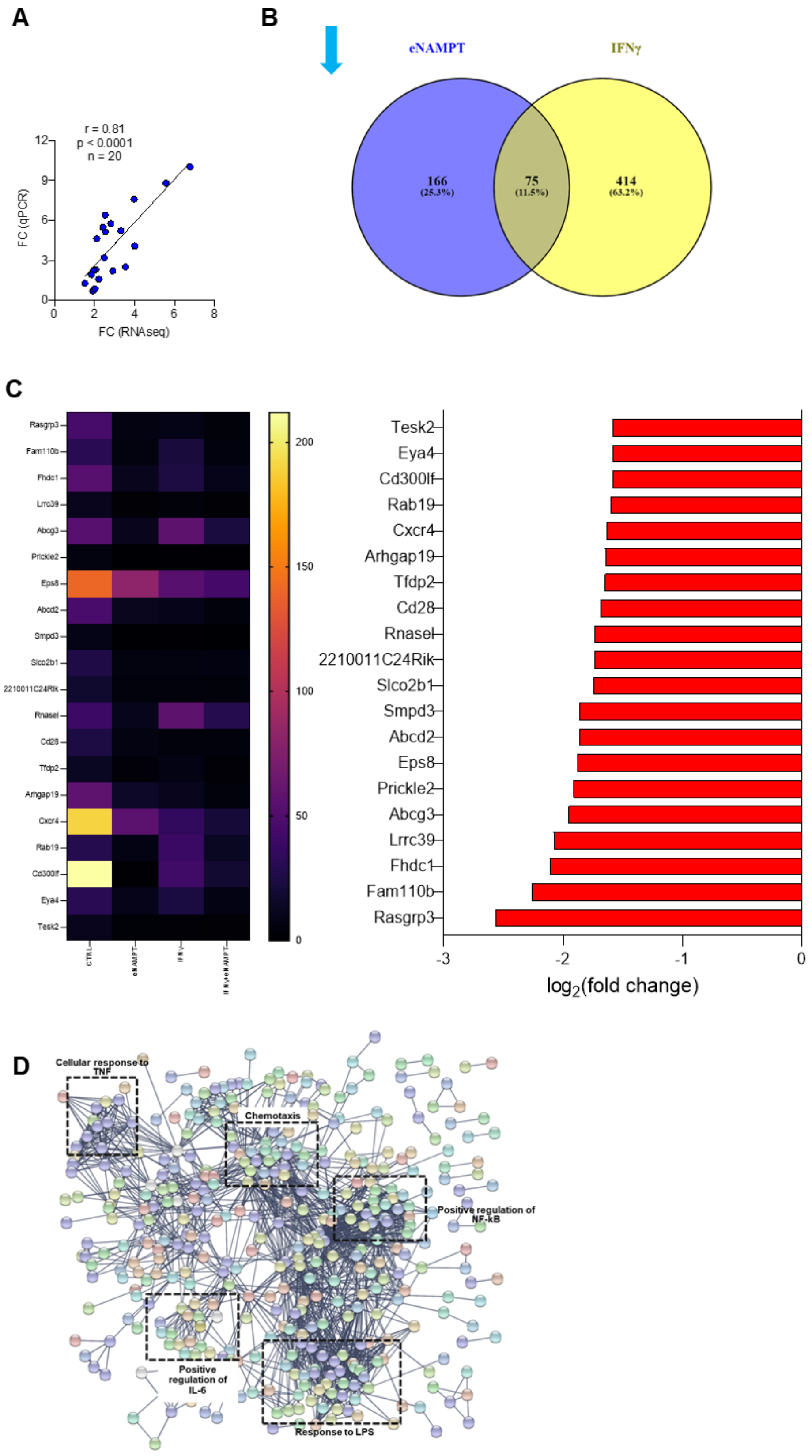

Supplementary Figure 2 related to Figure 2, Genes down-regulated by eNAMPT treatment. (A) Correlation between fold-changes of PECs treated with eNAMPT of 20 selected genes using qPCR

(2−ΔΔCt) and RNseq (log2 fold-change). Data are mean ± SE (Pearson's correlation). (**B**) Venn diagram of the relationship between eNAMPT- and IFNγ-regulated genes (FDR ≤ 0.1 and log2 fold-change > 1) (**C**) Heat map and histogram representation depicting the expression of downregulated genes in PECs after eNAMPT stimulation (Padj ≤ 0.01 and log2-fold change ≥ 2). (**D**) Protein–protein interaction (PPI) network built with all 407 significantly eNAMPT-upregulated genes using STRING. Further functional analysis was performed with DAVID and significantly enriched categories are highlighted.

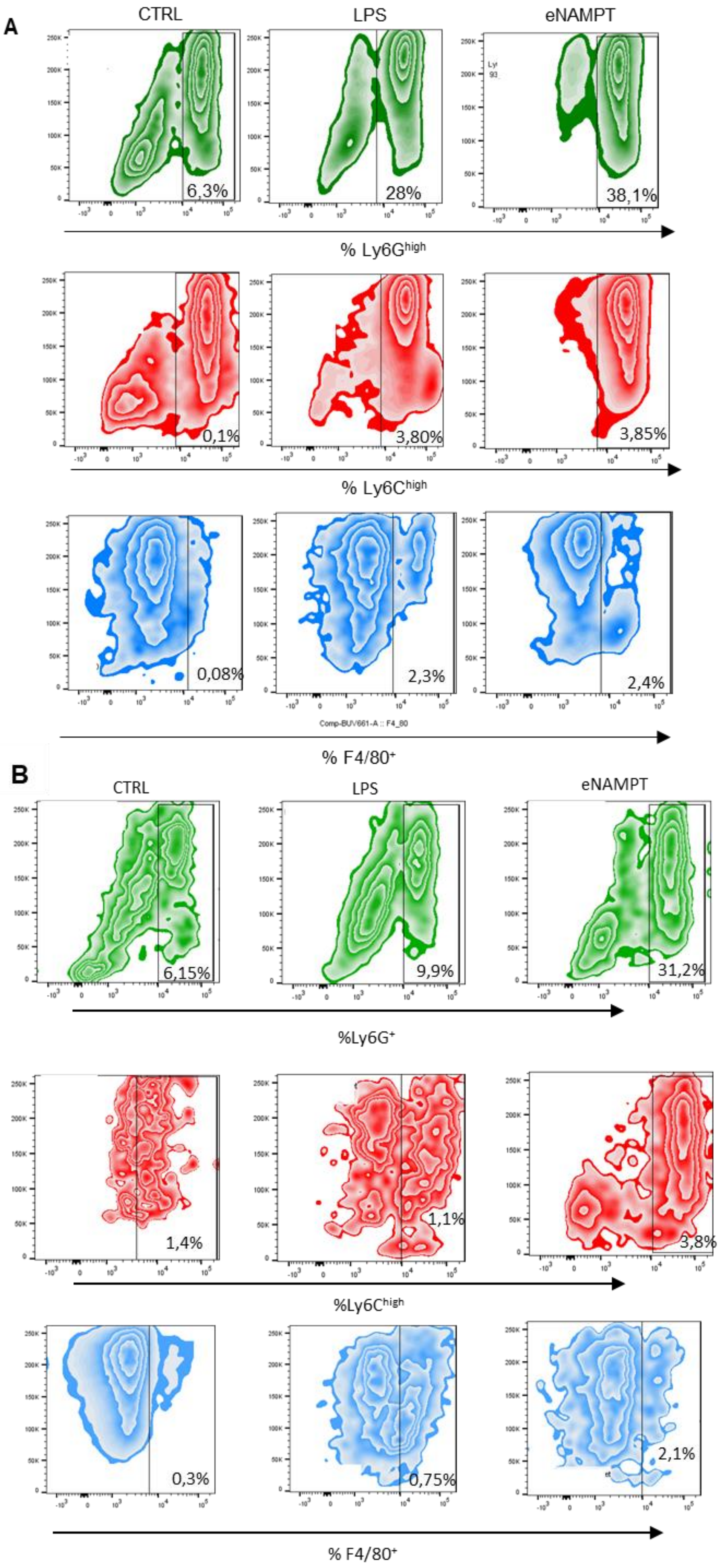
A
CTRL
LPS
eNAMPT
6,3%
28%
38,1%
% Ly6Ghigh
0,1%
3,80%
3,85%
% Ly6Chigh
0,08%
2,3%
2,4%
Comp-BUV661-A :: F4_80
% F4/80+
B
CTRL
LPS
eNAMPT
6,15%
9,9%
31,2%
%Ly6G+
1,4%
1,1%
3,8%
%Ly6Chigh
0,3%
0,75%
2,1%
% F4/80+

Supplementary Figure 3, related to Figure 3, Contour plot of immune cells isolated from pouches. Representative contour plot of FACS analysis using FlowJo V10 of immune cells isolated from air pouches from wild-type (**A**) or TLR4-KO mice (**B**).

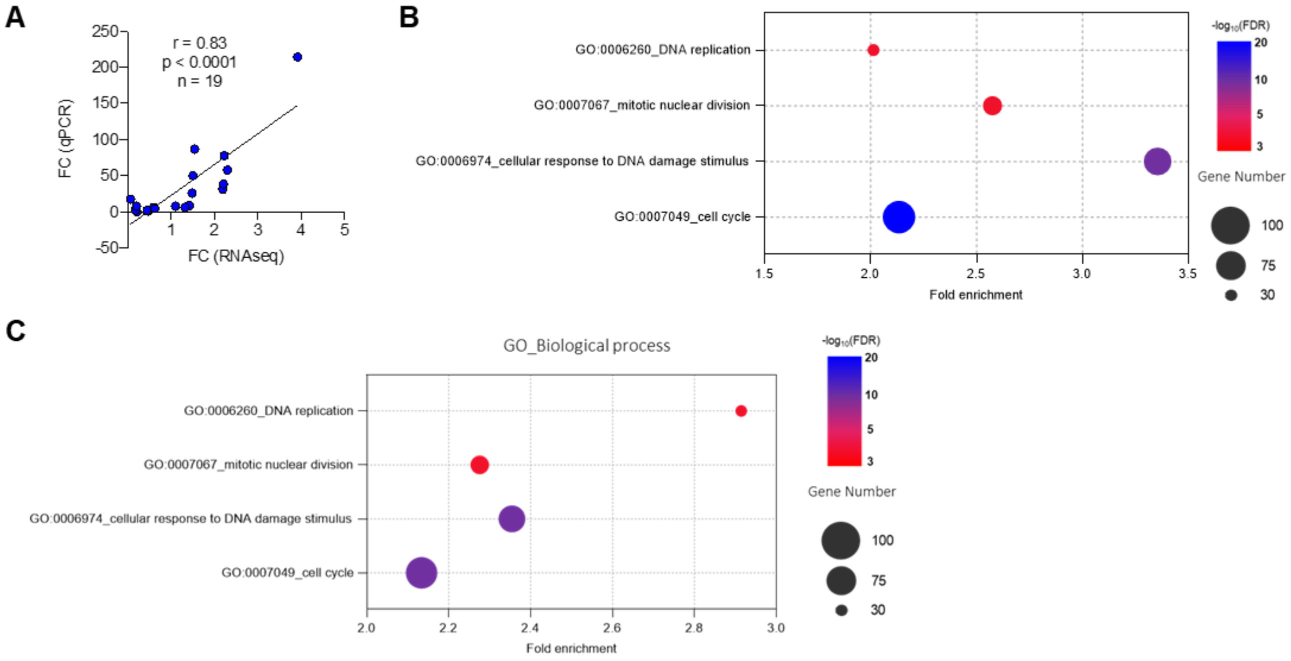

Supplementary Figure 4, related to Figure 4, Pathways downregulated by eNAMPT and IFNγ treatment. (**A**) Correlation between fold-changes of PECs treated with eNAMPT+IFNγ of 19 selected genes using qPCR (2−ΔΔCt) and RNseq (log2 fold-change). Data are mean ± SE (Pearson's correlation); (**B**) Gene ontology analysis of IFNγ-downregulated genes; (**C**) Gene ontology analysis of eNAMPT+IFNγ-downregulated genes.

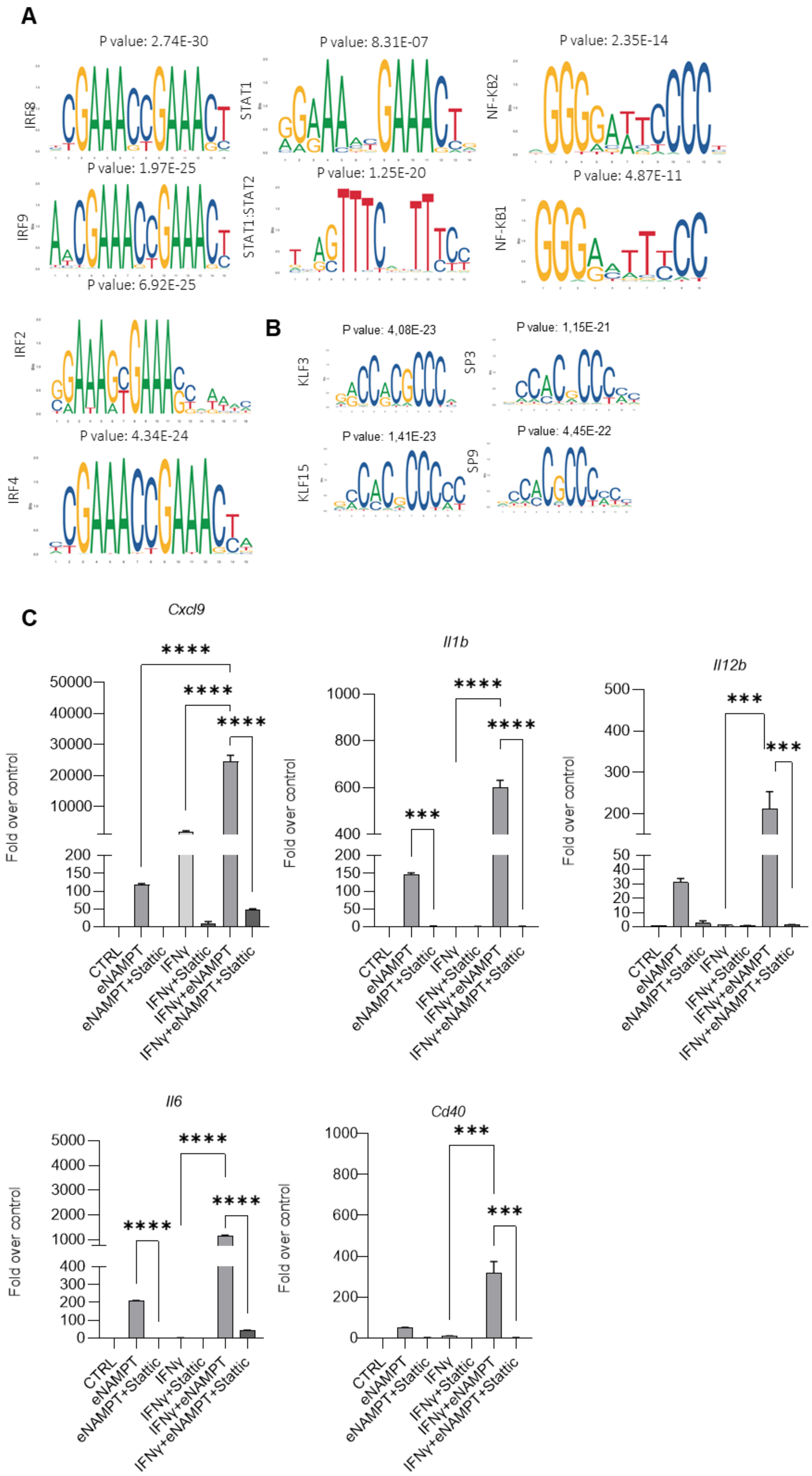
A
P value: 2.74E-30
IRF8
P value: 8.31E-07
STAT1
P value: 2.35E-14
NF-KB2
P value: 1.97E-25
IRF9
P value: 1.25E-20
STAT1:STAT2
P value: 4.87E-11
NF-KB1
P value: 6.92E-25
IRF2
P value: 4.34E-24
IRF4
B
P value: 4,08E-23
KLF3
P value: 1,15E-21
SP3
P value: 1,41E-23
KLF15
P value: 4,45E-22
SP9
C
Cxcl9
Il1b
Il12b
Fold over control
CTRL
eNAMPT
eNAMPT+Stattic
IFNγ
IFNγ+Stattic
IFNγ+eNAMPT
IFNγ+eNAMPT+Stattic
Il6
Cd40

Supplementary Figure 5, related to Figure 5, the cytokine eNAMPT enhances IFNγ-induced STAT1 and STAT3 phosphorylation. (**A**) Patterns of TF motif enrichment within the promoters of the IFNγ in PECs (**B**) Patterns of TF motif enrichment within the promoters of the eNAMPT+IFNγ in PECs. Patterns highlighted in the single treatments are not depicted. (**C**) Gene expression changes PECs treated with eNAMPT (500 ng/ml) and/or IFNγ (200 U/ml) for 4 hours, in presence or absence of Stattic (3μM). Mean ± S.E.M. of 2 independent experiments.
